# Identification of cerebrospinal fluid and serum metabolomic biomarkers in first episode psychosis patients

**DOI:** 10.1038/s41398-022-02000-1

**Published:** 2022-06-03

**Authors:** Pei Shang, Ada Man-Choi Ho, Maximilian Tufvesson-Alm, Daniel R. Lindberg, Caroline W. Grant, Funda Orhan, Feride Eren, Maria Bhat, Göran Engberg, Lilly Schwieler, Helena Fatouros-Bergman, Sophie Imbeault, Ryan M. Iverson, Surendra Dasari, Fredrik Piehl, Simon Cervenka, Carl M. Sellgren, Sophie Erhardt, Doo-Sup Choi

**Affiliations:** 1 Department of Molecular Pharmacology and Experimental Therapeutics, Mayo Clinic College of Medicine and Science, Rochester, MN USA; 2 Department of Neurology, First Hospital of Jilin University, Changchun, China; 3 Department of Psychiatry and Psychology, Mayo Clinic College of Medicine and Science, Rochester, MN USA; 4 Department of Physiology and Pharmacology, Karolinska Institutet, Stockholm, Sweden; 5 Mayo Clinic MD/PhD Program, Mayo Clinic School of Medicine and Science, Mayo Clinic, Rochester, MN USA; 6 Neuroscience Program, Mayo Clinic College of Medicine and Science, Rochester, MN USA; 7 Research and Development, Innovative Medicines, Personalised Healthcare and Biomarkers, Translational Science Centre, Science for Life Laboratory, AstraZeneca, Solna, SE-17177 Sweden; 8 Center for Psychiatry Research, Department of Clinical Neuroscience, Karolinska Institutet & Stockholm Health Care Services, Region Stockholm, Stockholm, Sweden; 9 Division of Biomedical Statistics and Informatics, Mayo Clinic College of Medicine, Rochester, MN USA; 10 Unit of Neuroimmunology, Department of Clinical Neuroscience, Center for Molecular Medicine, Karolinska Institutet, Karolinska University Hospital, Stockholm, Sweden; 11 Department of Medical Sciences, Psychiatry, Uppsala University, Uppsala, Sweden

**Keywords:** Predictive markers, Schizophrenia

## Abstract

Psychotic disorders are currently diagnosed by examining the patient’s mental state and medical history. Identifying reliable diagnostic, monitoring, predictive, or prognostic biomarkers would be useful in clinical settings and help to understand the pathophysiology of schizophrenia. Here, we performed an untargeted metabolomics analysis using ultra-high pressure liquid chromatography coupled with time-of-flight mass spectroscopy on cerebrospinal fluid (CSF) and serum samples of 25 patients at their first-episode psychosis (FEP) manifestation (baseline) and after 18 months (follow-up). CSF and serum samples of 21 healthy control (HC) subjects were also analyzed. By comparing FEP and HC groups at baseline, we found eight CSF and 32 serum psychosis-associated metabolites with non-redundant identifications. Most remarkable was the finding of increased CSF serotonin (5-HT) levels. Most metabolites identified at baseline did not differ between groups at 18-month follow-up with significant improvement of positive symptoms and cognitive functions. Comparing FEP patients at baseline and 18-month follow-up, we identified 20 CSF metabolites and 90 serum metabolites that changed at follow-up. We further utilized Ingenuity Pathway Analysis (IPA) and identified candidate signaling pathways involved in psychosis pathogenesis and progression. In an extended cohort, we validated that CSF 5-HT levels were higher in FEP patients than in HC at baseline by reversed-phase high-pressure liquid chromatography. To conclude, these findings provide insights into the pathophysiology of psychosis and identify potential psychosis-associated biomarkers.

## Introduction

Schizophrenia is a detrimental illness with a lifetime prevalence of 0.7% and is associated with increased mortality and a shortened lifespan. The disorder is characterized by positive (hallucinations, delusions, confused thought, and disorganized speech) and negative symptoms (blunting of affect, apathy, anhedonia, reduced social drive, loss of motivation, lack of social interest) as well as a loss of cognitive functions (with regard to attention, working memory, verbal learning/memory, and executive functions). Schizophrenia symptoms usually first appear in adolescence or early adulthood, and as early detection and care of subjects with psychosis significantly improve clinical outcomes, it is essential to identify afflicted persons at an early stage [1]. The diagnosis of schizophrenia is based entirely on the subjective interpretation of psychiatric symptoms presented by the patient. Symptoms alone are of marginal predictive significance, thus biological markers predicting psychosis or schizophrenia would be of immense value. Identifying biomarkers associated with psychosis would also assist in disclosing the underlying pathophysiology of schizophrenia spectrum disorders.

For long, the pathophysiology of schizophrenia has been attributed to brain dopaminergic dysfunctions [2]. However, the symptom profiles show a high degree of heterogeneity between patients, indicating a broad range of pathophysiological mechanisms. Expanding﻿ our understanding of molecular mechanisms in schizophrenia is thus crucial for disease prevention and treatment.

Metabolomics is the comprehensive analysis of small molecule metabolites in biological samples and has emerged as an evolving technology with a promising approach for identifying potential biomarkers for diagnosis, monitoring, prediction, or prognosis. In line with this, we recently identified an index of baseline biomarkers with high positive predictive values for acamprosate treatment-response in alcohol-dependent patients [3]. Metabolomics is further suggested to hold promise to advise on the practice of precision medicine [4], which is an area of great need for schizophrenia [5]. In schizophrenia research, metabolomics has been applied to plasma in numerous studies [6]. To our knowledge, few studies have focused on the brain in this regard; only one study has analyzed metabolomics in the postmortem prefrontal cortex of patients with schizophrenia and another in the cerebrospinal fluid (CSF) of patients prodromal for psychosis [7]. We utilized both CSF and serum samples, which represent the central nervous system (CNS) and the peripheral circulation, respectively. Thus, studying their metabolomes jointly would identify potential psychosis-associated metabolic pathways and biomarkers in the CNS and the peripheral system independently as well as interactively. It is also possible to reveal serum metabolomic biomarkers that represent CNS metabolic changes via the comparison with the CSF metabolome, hence more cumbersome CSF collection could be exempted in the future.

This study utilized ultra-high pressure liquid chromatography coupled with time-of-flight mass spectroscopy (UPLC-ToF/MS) to perform global metabolomic analyses on CSF and serum samples collected from patients presenting their first-episode psychosis (FEP) and at a follow-up assessment 18 months after. CSF and serum samples collected from healthy controls (HC) at corresponding time points were also analyzed. The objective was to identify clinically relevant biomarkers associated with psychosis, thereby providing important insights into the pathophysiology of schizophrenia. Furthermore, we aimed to identify putative catalogs of metabolic biomarkers that may aid in diagnosing schizophrenia spectrum disorders or be used for objectively monitoring disease progression. Using Ingenuity Pathway Analysis (IPA), we also identified candidate signaling pathways that may contribute to the pathophysiology of schizophrenia spectrum disorders. Finally, by identifying changes in CSF metabolites, we validated the identified metabolites as markers for diagnosis and outcome predictability.

## Methods

### Subject recruitment and demographics

The Stockholm Regional Ethics Committee (Dnr 2010/879-31/1) approved the protocol of this study. All participants had given written informed consent before any procedures according to the Declaration of Helsinki. FEP patients and HC were recruited from four psychiatric clinics under the Stockholm City Council for the Karolinska Schizophrenia Project (KaSP). At recruitment, all FEP patients met the DSM-IV criteria for psychotic disorders while HC were deemed healthy by medical history and examination with no history and no first-degree relatives of psychotic illness. Participants with a history or current use of illegal substances (e.g., opioids, cocaine, amphetamine, cannabis) were excluded but were allowed to use nicotine products (cigarettes or snuff; Table 1 and Supplementary Table S1) and alcohol. Psychiatric evaluation, cognitive testing, and CSF and blood sampling were performed on all participants at the time of recruitment (baseline) and for those who returned at follow-up 18 months later. This study included 47 FEP patients and 21 HC, in which 25 FEP patients and all HC had completed all components of both visits and were selected for the untargeted metabolomics profiling experiment, while the rest were included as an extended cohort for targeted metabolomics validation. Notably, about half of FEP patients had begun antipsychotic treatment (olanzapine, aripiprazole, risperidone, flupentixol, quetiapine, or haloperidol) prior to baseline assessment for no more than 30 days (Supplementary Table S2). Details of diagnostic criteria, exclusion criteria, and cognitive testing are described in Supplementary methods.

**Table 1:** Baseline subject demographics and clinical characteristics for untargeted metabolomics.

Characteristics	Healthy controls (HC)	First-episode Psychosis (FEP)	*p*-value
*n*	21	25	-
Gender (male/female)	8/13	13/12	0.387
BMI (mean ± SEM)	22.84 ± 0.61^a^	23.00 ± 0.66	0.886
Age (mean ± SEM)	25.81 ± 1.34	31.40 ± 1.96	0.028
Nicotine (*n*; %)	1 (5.0%)^b^	5 (22.7%)^b^	0.187
DUP (months; mean ± SEM)	0	16.45 ± 4.54^c^	-
PANSS (mean ± SD)			
Positive Symptoms	-	19.72 ± 6.18	-
Negative Symptoms	-	17.48 ± 8.50	-
General	-	38.04 ± 11.61	-
Total	-	75.24 ± 23.25	-
Levels of Functioning			
CGI Score (mean ± SD)	-	4.48 ± 1.16	-

*p*-values between gender and nicotine difference are calculated with Fisher’s exact test. *p*-values between age and BMI are calculated with binomial logistic regression.

*BMI* body mass index, *DUP* duration of untreated psychosis, *PANSS* positive and negative syndrome scale.

^a^Based on 19 HC subjects.

^b^Based on 20 HC subjects and 22 FEP subjects.

^c^Based on 22 FEP subjects.

### CSF and serum sample collection

CSF was collected using standard lumbar puncture protocols between 07:45 and 13:15 (overnight fasting in about 55% of subjects) with peripheral venous blood was sampled using standard venipuncture techniques at the same time as the CSF collection. Details of sample collection and processing procedures are described in Supplementary methods.

### Untargeted metabolomic analysis

Untargeted metabolomic analysis of the CSF and serum samples was performed by UPLC- TOF/MS (Agilent 1290 Infinity UHPLC coupled with 6550 iFunnel Q-TOF, Agilent Technologies, CA, USA) at the Mayo Clinic Metabolomics Core [8]. UPLC-TOF/MS method, mass spectrometry data processing, and protein identification details are described in Supplementary methods.

### Pathway analysis

Pathway analysis was performed on metabolites significantly different between groups at baseline and between time points by the Ingenuity Pathway Analysis program (IPA; Qiagen, Redwood City, CA, USA). Metabolite identifiers from various metabolite databases were submitted to IPA to maximize metabolite recognition (Supplementary methods). Significantly enriched pathways/processes were considered at Benjamini-Hochberg adjusted *p* < 0.05.

### Measurements of serotonin, 5-hydroxyindoleacetic acid, and tryptophan levels in baseline CSF

To follow up with our major findings, we measured the levels of serotonin (5-HT) and 5-hydroxyindoleacetic acid (5-HIAA) by reversed-phase HPLC and tryptophan by liquid chromatography with tandem mass spectrometry (LC-MS/MS) in baseline CSF samples of an extended cohort (Supplementary Table S1). Method details are described in the Supplementary methods.

### Statistical analyses

Subject demographics and basic clinical characteristics were compared between groups at baseline by binary logistic regression for continuous variables and Fisher’s exact test for categorical variables. Psychiatric scales and cognitive test results of FEP patients were compared between baseline and 18-month follow-up by paired *t-*tests. GraphPad Prism 8 (GraphPad Software, La Jolla, CA, USA) was used to perform these analyses.

The overall study design was carried out to identify metabolites that were significantly altered in FEP patients compared to HC or at follow-up compared to baseline (follow-up vs. baseline; Supplementary Fig. S1A). A metabolite was declared to be statistically significant at a false discovery rate (FDR)-adjusted *p* < 0.15 (i.e., FDR controlled at 15%) with a fold change that exceeded a certain empirical cutoff (Supplementary Tables S3 and S4).

One-way ANCOVA used to compare levels of serotonin and related metabolites measured by HPLC and LC-MS/MS between FEP patients and HC adjusting for age, antidepressant use and nicotine use. Statistical significance was set at Benjamini-Hochberg-adjusted *p* < 0.05. For the analysis of CSF tryptophan, 5-HT, and 5-HIAA levels in the HPLC cohort, outliers were identified and removed using the ROUT method (with Q set to 1%) in GraphPad Prism 8.

## Results

### Demographics and clinical characteristics of study participants

Subject demographics and clinical characteristics are summarized in Table 1 and Supplementary Table 1. FEP patients and HC did not differ in sex distribution, body mass index, or nicotine use in either the metabolomics or the extended cohort (*p* > 0.05). However, FEP patients were older than HC in the metabolomics and extended cohort (*p* < 0.05). FEP patients showed significant alleviations in symptoms and cognitive impairments at the 18-month follow-up compared to baseline, including reductions in PANSS positive, general and total scores, GAF symptom and function scores, and CGI scores, as well as elevations in BACS-SC, CPT-IP, and BVMT-R scores (Supplementary Table S5 and Supplementary Fig. S2).

### Metabolomic differences in CSF and serum between FEP patients and healthy controls

#### Baseline

**Fig. 1:**
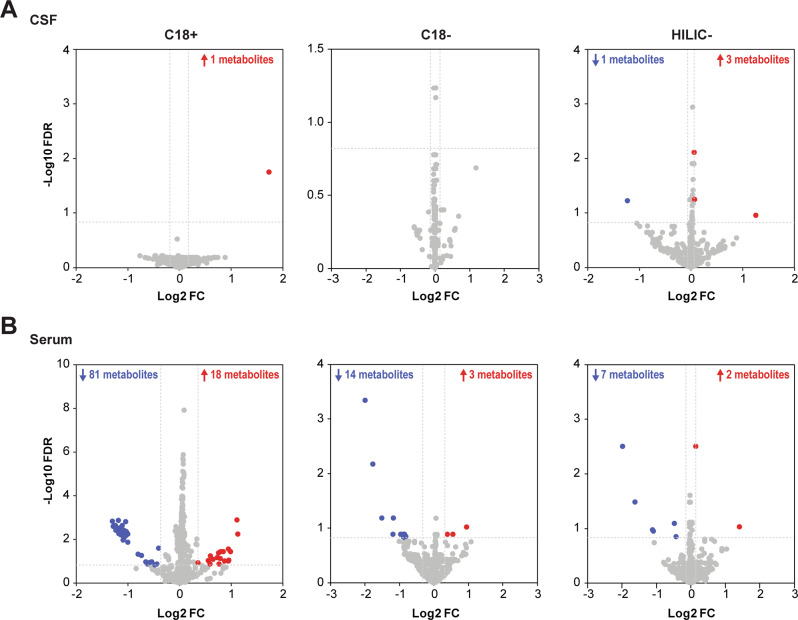
Volcano plots of psychosis-associated metabolites by detection modes. Each sample was subjected to all combinations of liquid chromatography (C18 or HILIC columns) and electrospray ionization (positive or negative) to maximize metabolite detection. Data for serum subjected to HILIC+ detection were discarded due to heterogeneities after normalization. **A** CSF metabolite changes in C18+, C18−, and HILIC− modes (from left to right). **B** Serum metabolite changes in C18+, C18−, and HILIC− modes (from left to right). Statistical significance was set at FDR < 0.15 (vertical gray lines) with a fold change threshold which was set at the top 5% of most variable metabolites comparing between baseline and follow-up in healthy controls for each mode (horizontal gray lines; Supplementary Table 3 and 4). Significantly down-regulated metabolites are colored in blue while significantly up-regulated metabolites are colored in red. (Abbreviations: C18, C18 column; CSF, cerebrospinal fluid; HILIC, hydrophilic interaction liquid chromatography column; +, positive electron ionization; −, negative electron ionization).

Eleven metabolites in CSF and 125 metabolites in serum were significantly different between HC and FEP patients at baseline, as defined by our FDR and fold change thresholds (Fig. 1; full lists in Supplementary Tables S6 and S7), out of which ten CSF and 32 serum metabolites were identified (Table 2). Among the ten identified CSF metabolites, two were detected in different UPLC-TOF/MS modes (5-HT and pyriculol), thus resulting in eight non-redundant identified CSF metabolites. No identification redundancy was found among the identified serum metabolites. Since no metabolites were considered age-related, all these metabolites could be considered psychosis-associated metabolites. No overlap of identified metabolites was detected in CSF and serum at baseline (Supplementary Fig. S1B). The list of psychosis-associated CSF metabolites mainly consists of 5-HT, acanthicifoline, guvacoline, pyriculol, athamantin, citric acid, and acetylene dicarboxylate. Notably, the levels of 5-HT, citric acid, acanthicifoline, guvacoline, and pyriculol were more than doubled in the CSF of FEP patients compared to HC. The catalog of psychosis-associated serum metabolites mainly consisted of drugs and their metabolites, including norathyriol, tetracaine, nalorphine, lodoform, and gabapentin. Of note, the levels of N-docosahexaenoyl GABA were higher in the sera of FEP patients than in the HC.

**Table 2:** List of identified cerebrospinal fluid (CSF) and serum metabolites differed between first-episode psychosis patients (FEP) and healthy controls (HC) at baseline, i.e., psychosis-associated metabolites.

Sample	MS-mode	Metabolites	Log2FC (FEP/HC)	*p*-value	FDR-adjusted *p*-value
CSF	HILIC+	Acanthicifoline	1.660	2.87 × 10^–5^	2.82 × 10^−2^
		Guvacoline	1.595	2.42 × 10^−4^	4.76 × 10^−2^
		5-HT	1.592	5.54 × 10^−4^	6.06 × 10^−2^
		Pyriculol	1.270	3.03 × 10^−3^	1.30 × 10^−1^
		Athamantin	−0.542	5.17 × 10^−4^	6.06 × 10^−2^
	HILIC−	Citric acid	1.255	3.59 × 10^−3^	1.11 × 10^−1^
		Acetylene dicarboxylate	0.073	1.05 × 10^−3^	5.67 × 10^−2^
		Pyriculol	0.061	2.39 × 10^−5^	7.78 × 10^−3^
		RRG	−1.231	1.29 × 10^−3^	6.00 × 10^−2^
	C18+	5-HT	1.738	1.78 × 10^−5^	1.77 × 10^−2^
Serum	HILIC−	NDI	−0.478	1.41 × 10^−3^	8.15 × 10^−2^
		Norathyriol	−1.613	2.12 × 10^−4^	3.28 × 10^−2^
	C18+	Myxalamid A	1.134	1.25 × 10^−3^	5.88 × 10^−3^
		N-oleoyl methionine	0.951	1.09 × 10^−2^	2.86 × 10^−2^
		Val Val	0.802	1.60 × 10^−2^	3.75 × 10^−2^
		PE	0.758	1.90 × 10^−2^	4.29 × 10^−2^
		MND	0.737	3.59 × 10^−2^	7.00 × 10^−2^
		N-docosahexaenoyl GABA	0.653	4.52 × 10^−2^	8.41 × 10^−2^
		Trans-2,3,4-Trimethoxycinnamate	0.604	2.79 × 10^−2^	5.80 × 10^−2^
		S-Farnesyl Thioacetic Acid	0.594	8.38 × 10^−2^	1.36 × 10^−1^
		MG(0:0/18:0/0:0)	0.560	5.31 × 10^−2^	9.51 × 10^−2^
		Hexazinone	0.363	7.24 × 10^−2^	1.20 × 10^−1^
		estrone 3-sulfate	−0.402	9.53 × 10^−3^	2.63 × 10^−2^
		Nalorphine	−0.479	9.30 × 10^−2^	1.47 × 10^−1^
		GGC	−0.565	6.72 × 10^−2^	1.13 × 10^−1^
		Theobromine	−0.613	8.29 × 10^−2^	1.35 × 10^−1^
		(3Z)-Phytochromobilin	−0.799	2.30 × 10^−2^	4.97 × 10^−2^
		Tetracaine	−0.998	3.96 × 10^−3^	1.35 × 10^−2^
		Serratanidine	−1.027	1.16 × 10^−3^	5.56 × 10^−3^
		N-acetyl-S-farnesyl-L-Cysteine	−1.042	1.01 × 10^−3^	5.18 × 10^−3^
		Iodoform	−1.062	6.07 × 10^−4^	4.12 × 10^−3^
		Pseudopelletierine	−1.079	7.25 × 10^−4^	4.54 × 10^−3^
		Asp Lys Lys	−1.094	8.69 × 10^−4^	4.86 × 10^−3^
		Lysyl-Tyrosyl-Lysine	−1.106	5.34 × 10^−4^	3.84 × 10^−3^
		Pseudoargiopinin III	−1.132	4.86 × 10^−4^	3.73 × 10^−3^
		Arg Gln Ile	−1.139	4.86 × 10^−4^	3.73 × 10^−3^
		Thr Lys Lys	−1.161	2.85 × 10^−4^	3.09 × 10^−3^
		Cadiamine	−1.172	4.87 × 10^−4^	3.73 × 10^−3^
		Gabapentin	−1.236	1.96 × 10^−4^	2.40 × 10^−3^
	C18−	2-Thiopheneacrylic acid	0.560	3.17 × 10^−3^	1.29 × 10^−1^
		PS (13:0/12:0)	−0.870	3.36 × 10^−3^	1.29 × 10^−1^
		HDD	−1.510	3.69 × 10^−4^	6.54 × 10^−2^

Metabolites which showed the same directional changes in the healthy controls from baseline to follow-up were excluded due to potential aging factors.

*5-HT* serotonin, *RRG* Rhamnetin 3-(3”“-p-coumaryl-rhamnosyl)(1–3)-rhamnosyl-(1–6)-galactoside, *NDI* N-depyridomethyl-Indinavir, *PE* PE(20:5(5Z,8Z,11Z,14Z,17Z)/0:0), *MND* 17-Methyl-18-norandrosta-4,13(17)-dien-3-one, *GGC* Galalpha1-4Galbeta-Cer(d18:1/16:0); *HDD* 10-hydroxy-2E,8E-Decadiene-4,6-diynoic acid.

#### Follow-up

Seven metabolites in the CSF and 119 in serum significantly differed between HC and FEP patients at the follow-up occasion (Supplementary Tables S8 and S9). None of these metabolites were found to be different in both CSF and serum. Two CSF metabolites and 84 serum metabolites showed significant between-group differences at both time points (Supplementary Tables S8 and S9). The levels of citric acid and acetylene dicarboxylate were significantly higher in the CSF of FEP patients than HC at both time points, but such differences were not observed in serum. The levels of norathyriol and pseudoargiopinin III were significantly lowered at both time points in the sera of FEP patients.

**Metabolomic changes in CSF and serum between baseline and 18-month follow-up** Comparing metabolite levels at baseline to the follow-up occasion in the FEP patients, we observed significant changes in eight CSF metabolites and 19 serum metabolites (Supplementary Fig. S1C; Supplementary Tables S10 and S11). Twelve CSF and 78 serum metabolites were added to adjust for age between groups since they showed significant between-group differences at either time point or differed between groups at both time points but in opposite directions (i.e., increase at baseline but decrease at follow-up or vice versa; Supplementary Tables S12 and S13) and seven serum metabolites were excluded on the same grounds, resulting in a catalog of 20 CSF and 90 serum follow-up associated metabolites. Table 3 lists the follow-up associated metabolites in CSF and serum identified. The list of follow-up-associated CSF and serum metabolites mainly consists of lipids (e.g., 13-eicosenoic acid), pyridine alkaloids (e.g., acanthicifoline, guvacoline), and neurotransmitter derivatives (5-HT, N-docosahexaenoyl GABA). We provide heat maps of psychosis and follow-up associated metabolites in CSF/serum as Supplementary Fig. S3. Of note, the altered levels of unsaturated lipids, such as taurochenodeoxycholic acid, 15(S)-15-methyl PGF2α isopropyl ester, and N-methylundec-10-enamide were detected in serum from FEP patients at follow-up compared with baseline. At follow-up, the CSF levels of deoxymiroestrol were up-regulated in the FEP patients but down-regulated in HC (Supplementary Tables S14 and S15).

**Table 3:** List of identified follow-up-associated CSF and serum metabolites.

Sample	Comparison^a^	Mode	Metabolites	Log2FC	*p*-value	FDR-adjusted *p*-value
CSF	FEP_FU_/FEP_B_^b^	HILIC+	Deoxymiroestrol	0.220	1.30 × 10^−11^	6.38 × 10^−9^
			TAC	−0.556	6.25 × 10^−3^	6.40 × 10^−2^
		HILIC−	Lasiocarpine	−0.062	4.34 × 10^−13^	2.83 × 10^−10^
	FEP_B_/HC_B_	HILIC+	Acanthicifoline	1.660	2.87 × 10^−5^	2.82 × 10-^2^
			Guvacoline	1.595	2.42 × 10^−4^	4.76 × 10^−2^
			Athamantin	−0.542	5.17 × 10^−4^	6.06 × 10^−2^
		HILIC−	Pyriculol	0.061	2.39 × 10^−5^	7.78 × 10^−3^
			RRG	−1.231	1.29 × 10^−3^	6.00 × 10^−2^
		C18+	5-HT	1.738	1.78 × 10^−5^	1.77 × 10^−2^
	FEP_FU_/HC_FU_	HILIC−	Phenolphthalein	1.145	3.26 × 10^−3^	1.05 × 10^−1^
			DDQ	1.100	4.39 × 10^−3^	1.12 × 10^−1^
Serum	FEP_FU_/FEP_B_^b^	HILIC−	MPIE	0.579	9.65 × 10^−3^	6.72 × 10^−2^
			13-eicosenoic acid	0.540	2.07 × 10^−2^	1.02 × 10^−1^
			Nocodazole	−0.300	3.83 × 10^−2^	1.46 × 10^−1^
			TCA	−0.645	1.76 × 10^−2^	9.19 × 10^−2^
		C18+	ME	0.354	7.68 × 10^−3^	2.62 × 10^−2^
			Myxalamid A	−0.807	7.48 × 10^−4^	5.93 × 10^−3^
	FEP_B_/HC_B_	HILIC−	DMI	−0.478	1.41 × 10^−3^	8.15 × 10^−2^
		C18+	Myxalamid A	1.134	1.25 × 10^−3^	5.88 × 10^−3^
			Val Val	0.802	1.60 × 10^−2^	3.75 × 10^−2^
			PE	0.758	1.90 × 10^−2^	4.29 × 10^−2^
			MNDO	0.737	3.59 × 10^−2^	7.00 × 10^−2^
			DH-GABA	0.653	4.52 × 10^−2^	8.41 × 10^−2^
			TTMC	0.604	2.79 × 10^−2^	5.80 × 10^−2^
			MG(0:0/18:0/0:0)	0.560	5.31 × 10^−2^	9.51 × 10^−2^
			Hexazinone	0.363	7.24 × 10^−2^	1.20 × 10^−1^
			Nalorphine	−0.479	9.30 × 10^−2^	1.47 × 10^−1^
			GGC	−0.565	6.72 × 10^−2^	1.13 × 10^−1^
			Phytochromobilin	−0.799	2.30 × 10^−2^	4.97 × 10^−2^
		C18−	TPAA	0.560	3.17 × 10^−3^	1.29 × 10^−1^
			PS(13:0/12:0)	−0.870	3.36 × 10^−3^	1.29 × 10^−1^
			HDD	−1.510	3.69 × 10^−4^	6.54 × 10^−2^
	FEP_FU_/HC_FU_	HILIC−	AG-041R	0.469	1.15 × 10^−3^	5.33 × 10^−2^
			Urocanic acid	−0.629	2.08 × 10^−3^	7.42 × 10^−2^
		C18+	BHA	0.658	5.22 × 10^−2^	8.09 × 10^−2^
			Poloxalene	0.362	3.33 × 10^−3^	7.65 × 10^−3^
			IBGME	−0.392	2.40 × 10^−2^	4.19 × 10^−2^
			Mesuagin	−0.400	3.30 × 10^−3^	7.61 × 10^−3^
			PC(20:2(11Z,14Z)/0:0)	−0.457	6.89 × 10^−3^	1.42 × 10^−2^
			Lys Met Lys	−0.484	3.71 × 10^−2^	6.12 × 10^−2^
			DIQ	−0.560	1.00 × 10^−1^	1.42 × 10^−1^
			Theophylline	−0.580	3.84 × 10^−3^	8.53 × 10^−3^
			Hypoxanthine	−0.660	8.81 × 10^−2^	1.27 × 10^−1^
			Kurilensoside G	−0.693	1.12 × 10^−2^	2.17 × 10^−2^
			Myxalamid A	−0.721	5.15 × 10^−2^	8.03 × 10^−2^

^a^*B* baseline, *FEP* first-episode psychosis patients, *FU* follow-up; *HC* healthy controls.

^b^Metabolites which showed the same directional changes in the healthy controls from baseline to follow-up (HC_FU_ vs. HC_B_) were excluded due to potential aging factors.

*TAC* Trans-3-Aminocyclopentane-1-carboxylic acid, *RRG* Rhamnetin 3-(3”“-p-coumaryl-rhamnosyl)(1–3)-rhamnosyl-(1–6)-galactoside, *5-HT* serotonin, *DDQ* 2,4-Diamino-6,7-dimethoxyquinazoline, *MPIE*15(S)-15-methyl PGF2α isopropyl ester, *TCA* Taurochenodeoxycholic acid, *ME* N-methylundec-10- enamide, *DMI* N-depyridomethyl-Indinavir, *PE* PE(20:5(5Z,8Z,11Z,14Z,17Z)/0:0), *MNDO* 17-Methyl-18-norandrosta-4,13(17)-dien-3-one, *DH-GABA* N-docosahexaenoyl GABA, *TTMC* Trans-2,3,4− Trimethoxycinnamate, *GGC* Galalpha1-4Galbeta-Cer(d18:1/16:0), *TPAA* 2-Thiopheneacrylic acid, *HDD* 10- hydroxy-2E,8E-Decadiene-4,6-diynoic acid, *BHA* Bis (2-hydroxypropyl) amine, *IBGME* Isobutyrylglycine methyl ester, *DIQ* 1,3-Dimethyl-6,8-isoquinolinediol.

### Correlations between 5-HT levels and symptoms and cognitive improvements

5-HT was identified as one of the most significantly differing psychosis-associated metabolites and is further well-recognized for its implications in schizophrenia. We further performed correlation analyses between symptoms or cognitive scores and 5-HT levels in the CSF or serum at baseline or follow-up. We observed negative correlations between Brief Assessment of Cognition in Schizophrenia symbol coding subtest scores and both baseline CSF (*ρ* = −0.303, *p* = 0.041) and serum 5-HT levels (*ρ* = −0.289, *p* = 0.051; Supplementary Tables S16–S19). We also found the change in serum 5-HT levels showed non-significant negative correlations with the changes in PANSS positive subscale (*ρ* = −0.374, *p* = 0.066) and GAF functioning subscale scores (*ρ* = −0.388, *p* = 0.067), while the change in CSF 5-HT levels were negatively correlated with PANSS negative (*ρ* = −0.390, *p* = 0.054), general (*ρ* = −0.425, *p* = 0.034), and total scores (*ρ* = −0.448, *p* = 0.025) as well as MSCEIT-ME score (*ρ* = −0.472, *p* = 0.020; Supplementary Tables S20 and 21). However, these correlations were not statistically significant after multiple testing correction.

### Pathway analysis of psychosis and follow-up associated metabolites

To identify whether the psychosis-associated and follow-up-associated metabolites are enriched in certain pathways, we employed IPA to perform enrichment analysis as previously reported [9]. Top enriched pathways in CSF psychosis-associated metabolites included “serotonin and melatonin biosynthesis”, “5-HT degradation”, and “5-HT receptor signaling pathways” (B-H *p* > 0.05; Supplementary Fig. S4). In serum psychosis-associated metabolites, top enriched pathways included “glutamate dependent acid resistance”, “phosphatidylethanolamine biosynthesis”, “tyrosine biosynthesis”, and “tyrosine biosynthesis” (B-H *p* > 0.05; Supplementary Fig. S5). Note that “tryptophan degradation to 2-amino-3-carboxymuconate semialdehyde” was predicted to be downregulated despite not statistically significant (B-H *p* > 0.05). Some significantly enriched biological functions overlap between CSF and serum psychosis-associated metabolites, such as “lipid metabolism”, “neurological disease”, and “cell death and survival” (Supplementary Figs. S4, S5).

Top enriched pathways in CSF follow-up-associated metabolites included “5-HT and melatonin biosynthesis”, “γ-aminobutyrate degradation”, “tyrosine degradation”, “tyrosine biosynthesis”, and “L-DOPA degradation” (B-H *p* > 0.05; Supplementary Fig. S6). In serum follow-up-associated metabolites, top enriched pathways include “tyrosine biosynthesis”, “4-aminobutyrate degradation”, “glutamate degradation via 4-aminobutyrate”, “phosphatidylethanolamine biosynthesis”, and “ceramide biosynthesis (B-H *p* > 0.05; Supplementary Fig. S7). Top significantly enriched biological functions that overlapped between CSF and serum follow-up-associated metabolites included “psychological disorders”, “neurological disease”, “lipid metabolism”, “cell death and survival”, “inflammatory response”, and “free radical scavenging” (B-H *p* < 0.05; Supplementary Figs. S6, S7).

### CSF tryptophan, 5-HT, and 5-HIAA differences at baseline between FEP patients and HC in the extended cohort

**Fig. 2:**
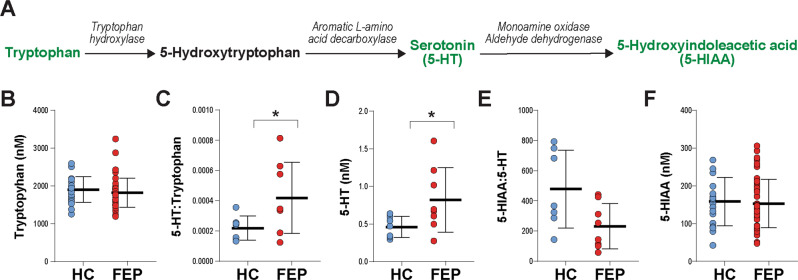
Serotonin and related metabolite levels in the baseline CSF samples of first-episode psychosis (FEP; *n* = 47) patients and healthy controls (HC; *n* = 21) identified by HPLC and LC-MS/MS. Tryptophan was detected in 68 samples (47 FEP and 21 HC), serotonin was detected in 15 samples (8 FEP and 7 HC), while 5-hydroxyindoleacetic acid (5-HIAA) was detected in 67 samples (47 FEP and 20 HC). **A** Serotonin synthesis pathway and enzymes involved are illustrated. Metabolites measured in the validation experiment are highlighted in green. Mean levels ± SD of (**B**) tryptophan, (**C**) serotonin-to-tryptophan ratio, (**D**) serotonin, (**E**) 5-hydroxyindoleacetic acid-to-serotonin ratio, and (**F**) 5-hydroxyindoleacetic acid are shown. Asterisks (*) represent differences between the FEP patients and HC after adjusting for age, antidepressant use, and nicotine use at *p* < 0.05. Standardized residuals are shown in Supplementary Fig. S8.

To validate the tryptophan-related metabolites, we examined tryptophan, 5-HT, and 5-HIAA levels in baseline CSF samples using HPLC in 47 FEP patients and 21 HC. Of the 68 samples available, tryptophan was reliably detected in 68 samples (47 FEP and 21 HC), 5-HT in 15 samples (8 FEP and 7 HC), and 5-HIAA in 67 samples (47 FEP and 20 HC). Consistent with metabolomics analysis, we found that baseline CSF 5-HT levels were higher in the FEP patients than the HC after adjusting for age, anti-depressant treatment, and nicotine use (*p* = 0.042; Fig. 2 and Supplementary Fig. S8). Also, as expected, the 5-HT/tryptophan ratio was higher in the FEP patients than the HC (*p* = 0.032; Fig. 2 and Supplementary Fig. S8). In contrast, tryptophan, 5-HIAA levels and 5-HIAA/5-HT ratio were similar between groups after adjusting for the aforementioned covariates (*p* > 0.05; Fig. 2 and Supplementary Fig. S8).

## Discussion

The present study provides comprehensive metabolomics signatures in both CSF and serum associated with psychosis and 18 months follow-up from FEP patients with and without antipsychotic treatment. As we understand, this is the first study attempts to identify the metabolomics profile of schizophrenia through a naturalistic treatment process without a specific-treatment approach or medication-based clinical trials. Previously, only a few studies have investigated the metabolomics of SCZ patients in a follow-up-based strategy. Here, we utilized a well-characterized cohort to analyze CSF and serum samples collected at both the onset of FEP and after 18 months, enabling us to study changes of metabolites that accompany the change in clinical symptoms. Our findings may reflect the changes of metabolites accompanied by the improvement of patient symptoms according to the PANSS score assessment. The stringent exclusion and inclusion criteria employed and our endeavor to match some key clinical variables between groups secured scientific rigor, however, some potential confounders were group-specific hence might affect our findings such as nicotine use and the use of antipsychotics and antidepressants [10]. We believe that our findings present several notable discoveries and include validation of existing knowledge.

One of the most striking findings reveals that increased CSF 5-HT levels at baseline appear to be associated with psychosis, confirmed by pathway analysis and validated in the extended cohort using a quantitative HPLC. The psychosis-associated and follow-up-associated metabolites identified were enriched in pathways related to 5-HT and melatonin (derived from 5-HT) biosynthesis, 5-HT receptor signaling, 5-HT degradation, and tryptophan degradation. An elevation in brain 5-HT was confirmed in our second cohort, where HPLC detection showed higher CSF levels in FEP patients at baseline. Notably, 5-HT levels were normalized to a level similar to the HC at 18-month follow-up, indicating that 5-HT changes are related to FEP onset. Since the FEP patients showed significant improvements in cognitive and psychological domains, these results implicate that 5-HT may be a potential biomarker for diagnosis and treatment progress monitoring. On the other hand, in the FEP patients, we did not observe altered CSF 5-HIAA, a 5-HT metabolite that is generally considered an appropriate predictive marker of schizophrenia [11–13]. In accordance with our findings of 5-HT and 5-HIAA levels in patients at baseline, the 5-HT/tryptophan ratio was increased, implicating a potential activation in the 5-HT biosynthesis at FEP.

Although numerous studies have focused on 5-HT’s role in the origin and progress of schizophrenia, most clinical studies analyzing the neurotransmitter (or 5-HIAA) in CSF or blood from schizophrenia patients were performed decades ago, and contradictory results were reported [14, 15]. The most prominent evidence for serotonergic dysfunction in the brains of schizophrenia patients comes from imaging, genetic association studies, and postmortem brain tissue studies on 5-HT receptors and transporters [14, 16, 17]. The role of 5-HT is also supported by the psychotomimetic effects of drugs such as lysergic acid diethylamide and psilocybin, both stimulating the 5-HT_2A_ receptor, as well as by the superior antipsychotic action of clozapine which is attributed to its relatively potent antagonistic effect on 5-HT_2A_ receptors [18, 19]^.^ 5-HT has indirectly shown to be implicated in negative symptoms by studies in treatment-resistant schizophrenia where antipsychotics were found to alleviate negative symptoms when combined with serotonin reuptake inhibitors [20]. Our present findings of changes in CSF 5-HT-related mechanisms together with the normalization of 5-HT during the improvement of psychotic symptoms as evident by HPLC validation and pathway analysis, further highlight the significance of 5-HT in the pathophysiology of psychosis.

A large body of clinical and experimental studies implicate dopamine, glutamate, GABA, and kynurenic acid in schizophrenia. In the present study, though, we see no striking changes in these neurotransmitters/modulators, or their related metabolites, neither at baseline nor at the follow-up. However, metabolites related to dopamine signaling were identified in the pathway analysis. The dopamine hypothesis of schizophrenia has become a long-standing theory for psychosis ever since dopamine receptor antagonists were recognized to improve positive symptoms [21].

Considering the significance of dopamine signaling in psychosis [22–24], it is thus not surprising that we identified metabolites related to tyrosine and L-DOPA synthesis or degradation as enriched pathways in CSF or serum at both time points (Supplementary Figs. S5–S7). Furthermore, mounting biochemical and genetic evidence points to an activation of the immune system as an underlying component in the pathology seen in schizophrenia [25–27]. Although no typical immune-markers of schizophrenia were found in our metabolomics, pathway analysis connected inflammatory pathways to the top ten associated pathways (Supplementary Figs. S5–S7). Notably, one must bear in mind that the failure of the untargeted global metabolomic method to significantly identify individual metabolites directly related to dopamine, glutamate, GABA, kynurenic acid, or the immune system may be partially due to technical limitations related to the sensitivity of the method.

We found that 13-eicosenoic acid was significantly increased in serum of FEP patients at follow-up, other lipids such as trans-3-aminocyclopentane-1-carboxylic acid, 10-hydroxy-2E,8E-decadiene-4,6-diynoic acid, taurochenodeoxycholic acid, and S-farnesyl thioacetic acid were also identified as disease- or follow-up associated metabolites. In line with previous studies, our data suggest an altered lipid metabolism in schizophrenia [10, 28–32]. A recent study suggested lipidomic abnormalities predated the onset of psychosis and serum lipidomic measurements could predict psychosis risk [33]. Of note, high levels of serum lipids, such as triglyceride and cholesterol, are correlated with the severity of symptoms and poor performance in the stable phase of schizophrenia, whereas membrane lipids, such as polyunsaturated fatty acids (PUFAs), are decreased in the acute phase [34]. The elevation of fatty acids, triglycerides, and cholesterol in patients’ sera at follow-up suggests an enhanced lipid metabolism and potential neuroinflammatory roles in the process of psychosis [34]. Of relevance to these results, lipidomic abnormalities was recently shown to predate the onset of psychosis, suggesting that serum lipidomic measures could be utilized to predict psychosis risk for individuals at a clinical high risk for psychosis [35].

We further identified significant alterations in several pyridine alkaloids in the CSF and sera of FEP patients, such as acanthicifoline, guvacoline, and theobromine. Interestingly, consumption of guvacoline, a betel alkaloid, has been identified to mitigate positive symptoms in schizophrenia patients [36]. Guvacoline could be hydrolyzed into guvacine which inhibits GABA uptake [37]. We observed increased citric acid levels in FEP patient’s CSF both at baseline and follow-up. Citric acid is a key metabolite in the tricarboxylic acid (TCA) cycle, which is one of the glucose metabolism pathways. Reduced activities of TCA cycle enzymes including aconitase, succinate thiokinase, and alpha-ketoglutarate dehydrogenase complex have been found in the postmortem dorsolateral prefrontal cortex in schizophrenia patients [38]. Impaired glucose metabolism was also found in postmortem brain tissues of schizophrenia patients [39]. Our findings suggest an altered glucose metabolism in the CNS of FEP patients, but further studies of TCA-related metabolites will confirm this postulation. *N*-docosahexaenoyl GABA, an unsaturated fatty acid that belongs to the *N*-acyl-GABA family and has been shown to increase cellular calcium levels [40], was observed to be higher in the sera of FEP patients in our study. Norathyriol level was lower in patients’ serum compared with HC at baseline. The precursor of norathyriol, mangiferin, prevents 6-hydroxydopamine-induced cell death and reduces pro-inflammatory mediators in a rat model of schizophrenia [41]. Drugs and their metabolites were also identified in our study including norathyriol, myxalamid A, nalorphine, and gabapentin. Of note, myxalamid A was reported to be up-regulated in FEP patients at baseline but down-regulated in follow-up compared with HC. 4-hydroxyproline, as an NMDAR-related amino acid was reported to be elevated in FEP patients and inferred to induce a compensatory response to glutamatergic hypofunction [42]. Our pathway analysis also identified a potential involvement of the 4-hydroxyproline pathway, which is associated with executive dysfunction in schizophrenia [43].

Several limitations need to be noted. Firstly, our FEP patients were, on average, somewhat older than our HC. Here, we endeavored to avoid false-positive findings due to potential age effects by removing age-related metabolites that were observed to be changed across time in HC. Additionally, we found no significant independent effect of age on our psychosis-associated CSF and serum metabolites using a multivariate model (Supplementary Tables S22 and S23). Secondly, FEP patients were prescribed different types and dosages of antipsychotics and possibly received various non-pharmacological therapies during the 18-month follow-up period, hence changes in metabolites at follow-up could not be attributed to a certain treatment. Thirdly, the metabolite identification method used in our untargeted metabolomics experiment depends on the number of mass spectrometry spectrums available for known metabolites, and some metabolites may share the same spectrums with others, hence the metabolomic signals presented in this study require validation of targeted metabolomics methods for more accurate identification and quantification. Fourthly, some metabolites may be degraded during storage even though stored frozen at −80 °C and only thaw upon metabolomic analysis, but due to the untargeted nature of our metabolomic profiling analysis, we cannot pinpoint which metabolites may have been degraded. For LC/MS and HPLC data, tryptophan is shown to be stable throughout several freezing and thawing cycles. When correlating the duration of storage (time in the freezer) with any of the metabolites, i.e., tryptophan, 5-HT and 5-HIAA, no significant correlations were observed. Fifthly, only about 40% of metabolites submitted were recognized by the IPA despite supplying as many types of identifiers as possible, in turn limiting the discovery of potential pathways and biological functions associated with FEP and psychosis recovery. Sixthly, our main finding of serotonin might be confounded by medications [44], nicotine and alcohol use [45, 46] as well as genetic variations [44]. Lastly, our replication sample was small. Larger independent samples would be required to replicate and validate our findings to establish a model, such as a threshold of 5-HT level, for psychosis diagnosis and prognosis.

To conclude, we identified several metabolites, such as 5-HT, 13-eicosenoic acid, and citric acid, as clinical meaningful potential psychosis metabolomic biomarkers in serum and CSF using untargeted metabolomics that may provide insights into FEP pathogenesis and help to assess prognosis. The most prominent findings include changes related to serotonergic neurotransmission found in the CSF. The psychosis and follow-up associated metabolites require replication and further clinical investigations to establish their validity as predictive biomarkers. Although beyond the scope of this study, future studies may take advantage of our results by establishing correlations between central and peripheral levels of significant metabolites. Such an approach may enable a rational biomarker analysis valuable for the diagnosis of psychotic disorders.

## Supplementary information

Supplemental Methods

Supplemental Tables S1-23

Supplemental Tables S24

Supplemental Figures
