# Supplementary material for: Identification of cerebrospinal fluid and serum metabolomic biomarkers in first episode psychosis patients
Source: Transl Psychiatry. 2022 Jun 3;12:229. doi: 10.1038/s41398-022-02000-1 (PMC9166796; doi:10.1038/s41398-022-02000-1)
Supplement: Supplementary file 1 — Supplemental Methods [file 41398_2022_2000_MOESM1_ESM.docx]

# Supplementary Methods

**Identification of cerebrospinal fluid and serum metabolomic biomarkers associated with psychosis at the first episode and 18 months later**

**Running Title**: Identifying metabolomic biomarkers for psychosis

Pei Shang, M.D.^1,2,^ ^11^, Ada Man-Choi Ho, Ph.D.^3,^ ^11^, Maximilian Tufvesson-Alm, Ph.D.^4,^ ^11^, Daniel R. Lindberg, Ph.D.^5,6^, Caroline W. Grant^1^_,_ Funda Orhan, Ph.D.^4^, Feride Eren^4^, Maria Bhat^7^, Göran Engberg, Ph.D.^4^, Lilly Schwieler, Ph.D.^4^, Helena Fatouros Bergman, Ph.D.^8^, Sophie Imbeault, Ph.D.^4^, Ryan M. Iverson^9^, Surendra Dasari, Ph.D.^9^, Fredrik Piehl, M.D. ,Ph.D.^10^, Simon Cervenka, M.D. ,Ph.D.^8^, Carl M. Sellgren, M.D. ,Ph.D.^4,^ ^8^, Sophie Erhardt, Ph.D.^4*^, Doo- Sup Choi, Ph.D.^1,3,6*^

**Table of Contents**

Materials and Methods3

1. Subjects3

1.1 Recruitment3

1.2 Subject medications5

1.3 Psychiatric and cognitive tests6

1. CSF and blood sampling and processing7
2. Untargeted quantitative large-scale metabolomic profiling8

3.1. Data analysis of untargeted metabolomic profiling9

3.2. Pathway analysis11

1. Targeted metabolomic analyses11

4.1. Analysis of tryptophan by liquid chromatography/mass spectrometry 11

4.2. Analysis of 5-HT and 5-hydroxyindoleacetic acid by HPLC12

4.3. Statistical analysis of targeted metabolomics12

References14

1. **Subjects**
   1. *Recruitment*

This study was approved by the Stockholm Regional Ethics Committee (Dnr 2010/879-31/1). All participants had given written informed consents before any study procedures according to the Declaration of Helsinki. Recruitment began in January 2011 and is ongoing. First-episode psychosis (FEP) patients and healthy controls (HC) were recruited for the Karolinska Schizophrenia Project (KaSP) at four psychiatric clinics under the Stockholm City Council. In this study, we included 47 FEP patients and 21 HC who provided their first biospecimen collection and cognitive assessments (baseline) between January 2011 and November 2015. At the time of recruitment, all FEP patients met the Diagnostic and Statistical Manual of Mental Disorders-IV (DSM-IV) criteria for psychotic disorders (eleven paranoid schizophrenia, four delusional disorders, four schizophrenia unspecified, 15 other schizophrenia, one acute and transient psychotic disorder, ten psychosis not-otherwise-specified, one schizoaffective syndrome, and one major depressive disorder [single episode, severe with psychotic features]). Exclusion criteria of FEP patients included the presence of neurologic or severe somatic illness, current use or history of drug abuse (e.g., opioids, cocaine, amphetamine, and cannabis) other than normal use of nicotine or alcohol, and co-existing neurodevelopmental abnormalities such as autism spectrum disorder. One patient was diagnosed with Hepatitis B and was therefore excluded from all biochemical analyses. HC were recruited by advertisement and were deemed healthy according to medical history and clinical examination. Exclusion criteria of controls included previous or current use of illegal drugs and first-degree relatives with psychotic illness. None of the HC subjects were on any psychiatric medication at the time of the study. Normal use of nicotine products (cigarettes or snuff) and/or alcohol was not an exclusion criterion in both groups. The absence of major brain abnormalities was confirmed by MRI in FEP patients and HC as evaluated by an experienced neuroradiologist at the MR Centre, Karolinska University Hospital, Solna, apart from one HC exhibiting signs of demyelinating disease but did not fulfill the criteria for a clinically isolated syndrome or multiple sclerosis and the clinical neurological exam was normal with no history of relevant neurological symptoms. CSF examination revealed oligoclonal bands, but no other abnormalities. Since the test results were similar to other controls, this subject was not excluded from the analysis.

Twenty-five FEP patients and 21 HC returned for reassessment and biospecimen collection approximately 18 months later between November 2012 and December 2017. Psychiatric reassessment was also performed as a part of the study protocol (17 paranoid schizophrenia, four delusional disorders, one disorganized schizophrenia, eleven schizophrenia unspecified, one other schizophrenia, one residual schizophrenia, one acute and transient psychotic disorder, five psychosis not-otherwise-specified, one schizoaffective syndrome, one major depressive disorder [single episode, severe with psychotic features], and four with no diagnosis. These subjects were included in the untargeted metabolomic profiling experiments since they had provided both baseline and follow-up CSF and serum samples (see **Section 3** and **Figure M1**). Baseline CSF samples of all subjects, including those who attended just the baseline visit, were further analyzed for serotonin-related metabolites (see **Section 4** and **Figure M1**).

| **Figure M1.** Numbers of subjects involved in each metabolomic experiment. |
| --- |
| 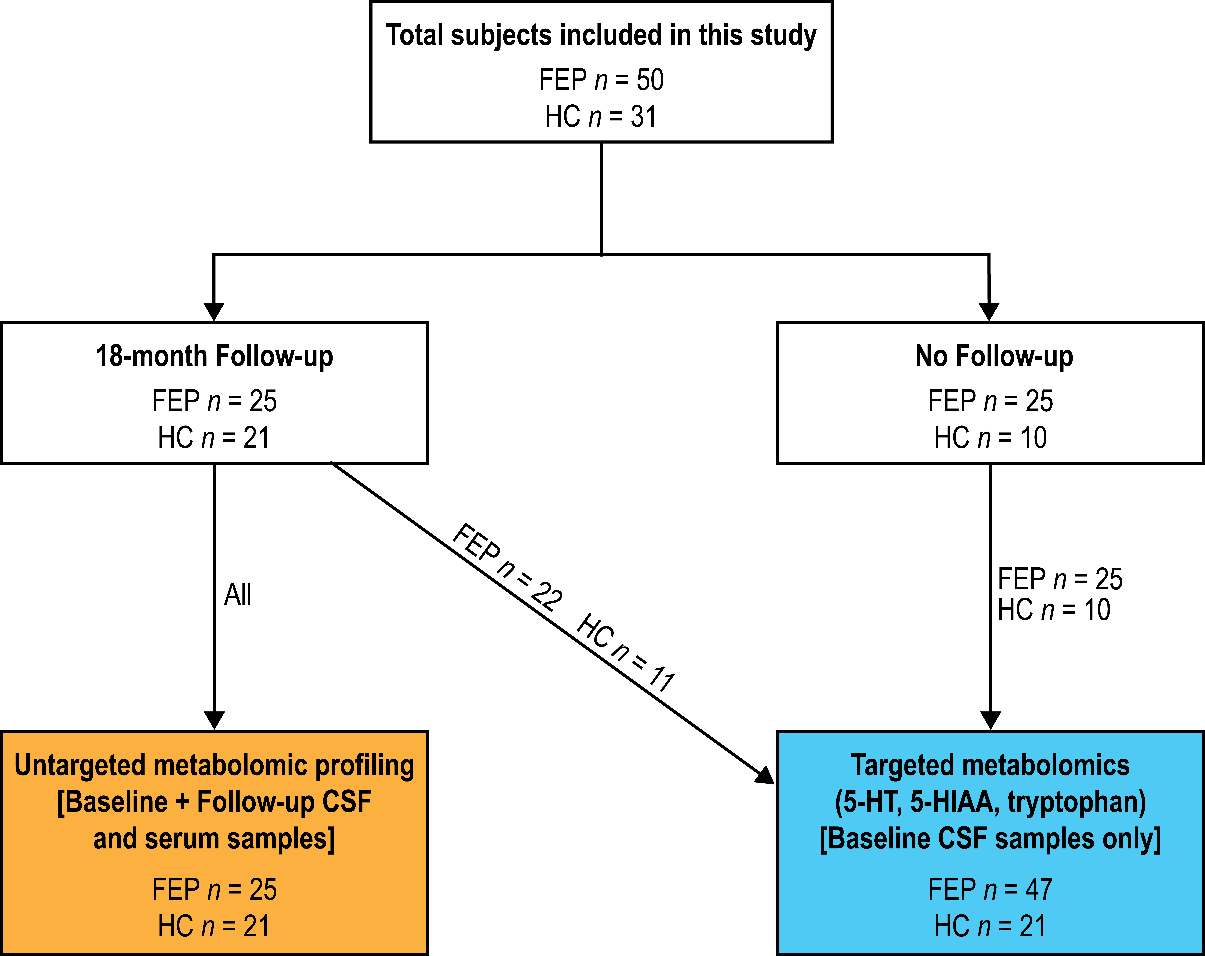 |

- 1. *Subject medications*

At baseline, 27 FEP patients had received psychotropic medications (olanzapine, aripiprazole, risperidone, flupentixol, quetiapine, or haloperidol) for a maximum of 30 days (mean ± s.e.m. = 12.8 ± 2.9 days; with one exception of 57 days) before biospecimen sampling (see **Supplementary Table S2**). Three patients were treated with two different antipsychotics. The rest of the 20 FEP patients had not used any kind of antipsychotic treatment before biospecimen sampling and were therefore considered as antipsychotics-naïve, but seven of these patients were treated with benzodiazepines or zopiclone and two with anti-depressants. A total of 14 FEP patients were treated with benzodiazepines or zopiclone and seven were treated with anti-depressants. For the 25 FEP patients included in the untargeted metabolomics study, twelve were antipsychotics-naïve and 13 had been treated with antipsychotics for a short time [mean time (± s.e.m.) 10 ± 2.9 days] before CSF sampling.

At follow-up, 15 patients were on antipsychotics and eight patients received antidepressants (information regarding antidepressant use is missing for one patient). Four patients had maintained antipsychotics-naïve through baseline to follow-up.

- 1. *Psychiatric and cognitive tests*

All subjects underwent a psychiatric evaluation at the time of recruitment and follow-up visit approximately 18 months later. Psychotic symptoms in patients were evaluated using the Positive and Negative Syndrome Scale for schizophrenia (PANSS), the Global Assessment of Function (GAF) scale, and the Clinical Global Impression (CGI) scale. PANSS, GAF, and CGI scores were obtained based upon a clinical interview including reports from family members and primary care hospital workers when necessary. The Mini-International Neuropsychiatric Interview was used to exclude previous or current psychiatric illness in the HC.

Cognitive function was assessed in all patients and HC using the Measurement and Treatment Research to Improve Cognition in Schizophrenia (MATRICS) Consensus Cognitive Battery (MCCB), which is designed specifically to measure cognition in the setting of schizophrenia (Green *et al.*, 2004). The cognitive domains assessed were speed of processing [Category Fluency Animal Naming, Trail Making Test (TMT): Part A, Brief Assessment of Cognition in Schizophrenia: Symbol Coding (BACS-SC)], attention/vigilance [(Continuous Performance Test-Identical Pairs (CPT-IP)], working memory [(Letter-Number Span (LNS), Wechsler Memory Scale: Spatial Span (WMS-III SS)], verbal learning [Hopkins Verbal Learning Test – Revised (HVLT-R)], visual learning [Neuropsychological Assessment Battery (NAB), Brief Visuospatial Memory Test-Revised (BVMT-R)] and social cognition [Mayer-Salovey-Caruso Emotional Intelligence Test-Managing Emotions branch (MSCEIT-ME)].

1. **CSF and blood sampling and processing**

Despite fasting was not required before biospecimen collection for subjects recruited between 2011 and 2013, the majority of subjects (*n* = 38; 24 patients and 14 controls) underwent lumbar puncture between 07:45 and 10:00 after overnight fasting. Owing to clinical routines, morning sampling was not possible in the remaining subjects (*n* = 30; 23 patients and 7 controls). The seven controls also underwent lumbar puncture during a similar time interval (10:30 to 13:15) to control for the potential confounding effects of collection time and fasting. Most subjects, i.e., those recruited after 2013, were instructed to avoid food intake and physical activity during the preceding 8 hours.

CSF sampling was performed using standard lumbar puncture protocols. Briefly, a disposable atraumatic needle (22G Sprotte, Geisingen, Germany) was inserted at the L4-5 level with all individuals in the right decubitus position. CSF (18 mL) was allowed to drip into a plastic test tube, protected from light. CSF supernatant from all subjects was divided into 10 aliquots that were frozen at −80 °C within 1 h of sampling following centrifugation (Sigma 5810R, Eppendorf, Hamburg, Germany at 3500 rpm [or 1438 *g*] for 10 min) to separate cells and supernatant, respectively.

Peripheral blood was collected in red top serum tubes using standard venipuncture techniques at the same time as CSF collection. Blood samples were centrifuged at 2900 rpm for 15 min within 1 h of collection to isolate plasma which was then stored at −80 °C until analysis.

1. **Untargeted quantitative large-scale metabolomic profiling**

Plasma samples were deproteinized with six times the volume of cold acetonitrile:methanol (1:1 ratio), kept on ice with intermittent vortexing for 30 minutes at 4ºC, then centrifuged at 18000 *g*. 13C6-phenylalanine (3 μL at 250 ng/μL) was added as an internal standard to each sample prior to deproteinization. The supernatants were divided into two aliquots and dried down for analysis on a Quadrupole Time-of-Flight Mass Spectrometer (Agilent Technologies 6550 Q-TOF) coupled with an Ultra High Pressure Liquid Chromatograph (1290 Infinity UHPLC Agilent Technologies). Profiling data were acquired under both positive and negative electrospray ionization conditions over a mass range of 50 – 1700 m/z at a resolution of 10,000-35,000 (separate runs), with a gas temperature of 225°C and a drying gas rate of 18 L/min. Metabolite separation was achieved using two columns of differing polarity, a hydrophilic interaction column (HILIC, ethylene-bridged hybrid 2.1×150 mm, 1.7 μm; Waters) and a reversed-phase C18 column (high-strength silica 2.1×150 mm, 1.8 μm; Waters). Both columns employed the same column temperature of 45°C and the same buffer system, consisting of Buffer A (20 mL acetonitrile, 10 mL 1M ammonium acetate, 2 mL formic acid, and 1968 mL water) and Buffer B (88 mL water, 10 mL 1M ammonium acetate, 2ml formic acid, and 1900ml acetonitrile). **Table M1** provides the LC gradient for the HILIC system, while **Table M2** provides the LC gradient for the C18 system. A total of four runs per sample were performed to give maximum coverage of metabolites. Samples were injected in duplicate, and a quality control sample, made up of a subset of samples from the study was injected several times during a run.

| **Table M1.** LC gradient for HILIC system. | | | |  | **Table M2.** LC gradient for C18 system. | | | |
| --- | --- | --- | --- | --- | --- | --- | --- | --- |
| **Time (min)** | **Buffer A (%)** | **Buffer B (%)** | **Flow rate (mL/min)** |  | **Time (min)** | **Buffer A (%)** | **Buffer B (%)** | **Flow rate (mL/min)** |
| 0.0 | 0.1 | 99.9 | 0.4 |  | 0.0 | 99.9 | 0.1 | 0.4 |
| 1.0 | 0.1 | 99.9 | 0.4 |  | 1.0 | 99.9 | 0.1 | 0.4 |
| 9.0 | 30.0 | 70.0 | 0.4 |  | 7.0 | 50.0 | 50.0 | 0.4 |
| 11.0 | 70.0 | 30.0 | 0.4 |  | 11.0 | 15.0 | 85.0 | 0.4 |
| 12.0 | 99.9 | 0.1 | 0.4 |  | 13.0 | 0.1 | 99.9 | 0.8 |
| 13.0 | 99.9 | 0.1 | 0.4 |  | 25.0 | 0.1 | 99.9 | 0.8 |
| 14.0 | 0.1 | 99.9 | 0.4 |  | 25.5 | 99.9 | 0.1 | 0.5 |
| 18.0 | 0.1 | 99.9 | 0.4 |  | 28.0 | 99.9 | 0.1 | 0.4 |

All raw data files obtained were converted to compound exchange file format using Masshunter DA reprocessor software (Agilent). Mass Profiler Professional (Agilent) was used for data alignment and to convert each metabolite feature (m/z × intensity × time) into a matrix of detected peaks for compound identification. An unsupervised principal component analysis, ANOVA, 3D plot and heat map, and a Partial Least Square discrimination analysis (PLS-DA) comparison between groups were obtained for analysis. This gave a list of accurate mass molecular weights of differentially expressed components that were run against the METLIN database to give putative identification (IDs). The list of components would have a putative ID or a mass (m/z) value depending on whether a match was found. Components that were assigned putative IDs were further examined by comparison to a purchased reference standard of the proposed compound. Mass accuracy of the Q-TOF method was <5ppm with retention time precision better than 0.2%. A fold change of 1.2 can be detected with a precision of 4%.

- 1. *Data processing and analysis of untargeted metabolomic profiling*

To filter low-intensity hits, a metabolite must have a peak intensity of at least 5000 in at least 75% samples to be eligible for downstream analyses. This primary intensity threshold was applied for all modes. R package *MetaboanalystR* was used for data normalization, differential expression analysis and visualization. In each mode, metabolites were row-wise normalized to a constant sum (*SumNorm*), log transformed, and scaled by mean centering (*MeanCenter*). Normalized data were analyzed by a multivariate approach such as Principal Component Analysis (PCA) to reveal data heterogeneity, groupings, outliers, and trends. Hierarchical clustering analysis (HCA) was done to reveal clustering between sample injections, group replicates, and multiple metabolite clusters that were correlated with different subsets of clinical variables.

The overall study design was carried out to identify metabolites that were significantly altered in FEP patients compared to HC (separately for baseline and follow-up) or changed at follow-up compared to baseline (follow-up vs. baseline; **Supplementary Figure S1A**). A metabolite was declared to be statistically significant at a false discovery rate (FDR) of 0.15 with a fold change that exceeded a certain empirical cutoff. For each sample type and acquisition mode, we first established a null distribution of fold changes by repeating five times of random assignment of each HC sample into one of two groups followed by a comparison between groups, then the minimum absolute fold change value which 95% of metabolites’ fold changes exceeded was taken as the cutoff (Serang *et al.*, 2014; **Supplementary Tables S3 and S4**). Psychosis-associated metabolites were defined as metabolites that were significantly different between FEP patients and HC at baseline, excluding metabolites that had significantly changed in HC at follow-up or shared the same changing direction with FEP patients (i.e., age-associated metabolites; **Supplementary Figure S1A**). It was assumed that these age-related metabolites which had altered after 18 months in HC could only be attributed to aging at physically-prime adulthood and the degrees of their alterations were the same in both subject groups. No age-related CSF metabolites were detected while five age-related serum metabolites were found (C25H12NO7, C23H32N11, C18H35N20O, C16H33N8O3, and ASP LYS LYS). Follow-up associated metabolites were defined as metabolites that were significantly different between baseline and follow-up in the FEP group with the inclusion of metabolites that were significantly different between groups only at baseline or only at follow-up or those that significantly differed between groups at baseline and follow-up by approximately the same magnitude but in opposite direction (i.e., increase at baseline, but decrease at follow-up, *or vice versa*) and the exclusion of age-associated metabolites (**Supplementary Figure S1A**).

- 1. *Pathway analysis*

Pathway analysis was performed on metabolites significantly different between groups at baseline and between time points by Ingenuity Pathway Analysis (IPA; Qiagen, Redwood City, CA, USA). IPA core analysis uses the right-tailed Fisher’s exact test to examine the likelihood of the overlap between a set of significant molecules from an experiment and a canonical pathway or biological process is due to random chance. Metabolite identifiers from the Kyoto Encyclopedia of Genes and Genomes (KEGG), Human Metabolome Database (HMDB), Chemical Abstract Service (CAS), and PubChem databases were submitted to IPA to maximize metabolite recognition. For CSF metabolites, we specified knowledge from the central nervous system and brain-and-immune-related cell types while keeping other settings as default. For serum metabolites, the default setting was applied which included knowledge from all listed tissues and cell types. Significantly enriched canonical pathways or biological processes were considered at Benjamini-Hochberg adjusted *p* < 0.05.

1. **Targeted metabolomic analyses**
   1. *Analysis of tryptophan by liquid chromatography/mass spectrometry (LC-MS/MS)*

All CSF samples were handled using a uniform protocol. Thus, samples were immediately centrifuged following sampling and stored frozen at −80 °C until analysis. Aliquoted cell-free CSF supernatant and standard samples were diluted 2× with internal standard (IS) solution in 5% formic acid and filtered at 3000 g for 60 min at 10 °C using 10 kDa Ultracel®-10 filter plates from Millipore. Following centrifugation, 7.5 μL of the filtrate was injected using a Waters Acquity HPLC system equipped with a SymmetryShield™ RP18 2.1 × 100 mm, 3.5 μm particle column. Detection was performed using a Waters Xevo TQ-S (Waters Corporation, Milford, USA) triple quadrupole mass spectrometer operating in positive ionization MS/MS configuration. The mobile phase was run at a flow rate of 300 μL/min and consisted of 2.1% formic acid in MilliQ water (A-phase) and 95% acetonitrile 0.1% formic acid (B phase) starting with 5% B for 2 min following gradient elution, the total run time of 10 min. Tryptophan was purchased from Sigma–Aldrich, St. Louise, USA. Calibration was performed using standards covering the range of the CSF concentration. Seven concentration points were used to establish a linear calibration curve for quantification. The calibration curve was plotted using the ratio of the analyte peak area over IS peak area after integration by Masslynx 4.1 software (Waters Corporation, Milford, USA). The retention time for tryptophan was 3.6 min.

- 1. *Analysis of 5-HT and 5-hydroxyindoleacetic acid by HPLC*

CSF was analyzed for 5-HT and 5-hydroxyindoleacetic acid (5-HIAA) with a reversed-phase HPLC system, including a pump (Bischoff Chromatography, Leonberg, Germany), an Agilent Eclipse XDB-C18 column (4.6×150 mm, Agilent Technologies, Inc., CA, USA), and a high- sensitivity analytical cell (ESA 5011; ESA Inc., Chelmsford, MA, USA) controlled by a potentiostat (Coulochem III; ESA Inc.) with an applied potential of -350 mV for detection. The mobile phase consisted of 55 mM sodium acetate buffer (pH 4.1, 10% methanol) with 1.16 mM octanesulfonic acid and 0.01 mM EDTA disodium Na_2_EDTA. The flow rate for the mobile phase was 0.7 mL/minutes. The signals from the detector were analyzed by Datalys Azur Software (Grenoble, France). The retention time of 5-HT and 5-HIAA was approximately 15 and 8 min, respectively.

- 1. *Statistical analysis of targeted metabolomics*

One-way ANCOVA was used to compare the levels of neurotransmitters and related metabolites measured by LC-MS/MS and HPLC between FEP patients and HC with adjustment of age, antidepressant use, and nicotine use. Statistical significance was set at Benjamini-Hochberg-adjusted *p* < 0.05. For the analysis of CSF tryptophan, 5-HT, and 5-HIAA levels in the HPLC cohort, outliers were identified and removed using the ROUT method (with Q set to 1%) in GraphPad Prism 8.

# References

Green MF, Nuechterlein KH, Gold JM et al. (2004) Approaching a consensus cognitive battery for clinical trials in schizophrenia: the NIMH-MATRICS conference to select cognitive domains and test criteria. *Biol Psychiatry* **56**(5), 301-307.

Chang AY, Antigoni ZL, Jenkins GD et al. (2017) Combining a nontargeted and targeted metabolomics approach to identify metabolic pathways significantly altered in polycystic ovary syndrome. *Metabolism-Clinical and Experimental* **71**, 52-63.

Dutta T, Chai HS, Ward LE et al. (2012) Concordance of changes in metabolic pathways based on plasma metabolomics and skeletal muscle transcriptomics in type 1 diabetes. *Diabetes* **61**(5), 1004-1016.

Dutta T, Kudva YC, Persson XMT et al. (2016) Impact of Long-Term Poor and Good Glycemic Control on Metabolomics Alterations in Type 1 Diabetic People. *J Clin Endocrinol Metab* **101**(3), 1023-1033.

Hale VL, Jeraldo P, Mundy M et al. (2018) Synthesis of multi-omic data and community metabolic models reveals insights into the role of hydrogen sulfide in colon cancer. *Methods* **149**, 59-68.

Serang O, Cansizoglu AE, Kall L et al. (2014) Nonparametric Bayesian evaluation of differential protein quantification. *J Proteome Res* **12**:4456-4565.

Trushina E, Dutta T, Persson XMT, Mielke MM, Petersen RC (2013) Identification of Altered Metabolic Pathways in Plasma and CSF in Mild Cognitive Impairment and Alzheimer's Disease Using Metabolomics. *Plos One* **8**(5),e63644.

Xyda SE, Vuckovic I, Petterson XM et al. (2020) Distinct Influence of Omega-3 Fatty Acids on the Plasma Metabolome of Healthy Older Adults. *J Gerontol A Biol Sci Med Sci* **75**(5), 875-884.
