# Supplementary material for: Identification of cerebrospinal fluid and serum metabolomic biomarkers in first episode psychosis patients
Source: Transl Psychiatry. 2022 Jun 3;12:229. doi: 10.1038/s41398-022-02000-1 (PMC9166796; doi:10.1038/s41398-022-02000-1)
Supplement: Supplementary file 2 — Supplemental Tables S1-23 [file 41398_2022_2000_MOESM2_ESM.pdf]

Supplementary Table S1. Demographics and clinical characteristics of the validation HPLC cohort.

	Healthy Controls	FEP Patients	*p*-value
n	21	47	
Gender (male/female)	11/10	28/19	0.606
BMI (kg/m^2; mean ± SEM)*	22.5 ± 0.66	23.4 ± 0.57	0.400
Age (years; mean ± SEM)	25.8 ± 1.12	30.9 ± 1.33	0.021
Nicotine (*n*; %)**	2 (10.0%)	11 (28.2%)	0.184
DUP (months; mean ± SEM)	0	10.6 ± 1.71	
PANSS (mean ± SEM)			
Positive	–	19.5 ± 0.79	
Negative	–	16.3 ± 1.10	
General	–	38.7 ± 1.72	
Total	–	74.5 ± 3.16	
Levels of Functioning (mean ± SEM)			
GAF symptoms	–	35.5 ± 1.86	
GAF Functioning	–	46.2 ± 1.36	

p-values between gender and nicotine difference were calculated with Fisher's exact test. *p*-values between age and BMI were calculated with binomial logistic regression.
*Information is missing for 5 HC and 2 FEP patients.
**Information is missing for 1 HC and 4 FEP patients.
Abbreviations: BMI, body mass index; DUP, duration of untreated psychosis; PANSS, positive and negative syndrome scale for schizophrenia; GAF, global assessment of function.

Supplementary Table S2.1. Medication information of exploratory metabolomic study cohort.

#FEP Patients	Neuroleptics (mg/day)	Benzodiazepine/ Zopiclone (mg/day)	Phenothiazine Derivatives (mg/day)	Antidepressant (mg/day)
Baseline				
1	–	Zopiclone (7.5)	–	–
2	–	–	–	–
3	Olanzapine (15*)	Oxazepam (5)	–	–
4	Olanzapine (10)	–	–	–
5	–	Oxazepam (15); Zopiclone (7.5)	–	–
6	Haloperidol (6); Olanzapine (5)	–	–	–
7	Aripiprazole (5)	–	Propiomazine (25)	–
8	Yes (No data on which medication type)	–	–	–
9	Risperidone (4)	Oxazepam (10)	–	Mirtazapine (30)
10	–	Zopiclone (7.5)	–	–
11	Olanzapine (30)	–	–	–
12	Olanzapine (10)	–	–	–
13	–	Zolpidem (10)	–	–
14	Quetiapine (50)	–	–	Venlafaxine (300)
15	–	Oxazepam (10); Zopiclone (7.5)	–	Paroxetine (20)
16	–	–	–	–
17	–	–	–	Citalopram (20)
18	–	–	–	–
19	–	–	–	–
20	Aripiprazole (15); Flupentixol (2)	–	–	Paroxetine (20)
21	–	–	–	–
22	Olanzapine (15)	Zopiclone (7.5)	–	–
23	–	Oxazepam (10)	–	–
24	Olanzapine (5)	–	–	–
25	Olanzapine (10)	Nitrazepam (5); Oxazepam (10)	Propiomazine (25)	–
Follow-up				
1	-			-
2	Aripiprazole (10*)			-
3	-			-
4	-			-
5	Haloperidol (6)			Yes
6	Haloperidol (4)			-
7	Aripiprazole (5)			-
8	-			-
9	Olanzapine (30); Clozapine (600)			Yes
10	-			-
11	Aripiprazole (15)			Yes
12	Olanzapine (10)			-
13	Quetiapine (800)			-
14	-			Yes
15	Quetiapine (50)			Yes
16	Aripriprazole (30)			-
17	Olanzapine (3)			Yes
18	-			-
19	Aripirazole (10)			-
20	Aripirazole (10)			Yes
21	-			-
22	-			-
23	Aripiprazole (5)			-
24	-			Yes
25	Aripiprazole (5)			N/A

Supplementary Table S2.2. Medication information of validation HPLC cohort.

#FEP Patients	Neuroleptics (mg/day)	Benzodiazepine/ Zopiclone (mg/day)	Phenothiazine Derivatives (mg/day)	Antidepressant (mg/day)
Baseline				
1	Olanzapine (15)	Oxazepam (5)	–	–
2	–	–	–	–
3	Olanzapine (10)	–	–	–
4	Olanzapine (10)/Quetiapine 600)	Oxazepam (10)	Propiomazin (25)/ Alimemazin (60)	–
5	Quetiapine (50)	–	–	Sertralin (50)
6	Olanzapine (10)	–	–	–
7	–	Oxazepam (15)/ Zopiklon (7.5)	–	–
8	Haloperidol (6)/Olanzapine (5)	–	–	–
9	Aripiprazole (5)	–	Propiomazin (25)	–
10	Olanzapine (15)	Oxazepam (15)	Propiomazin (25)	–
11	Aripiprazole (15)	–	–	–
12	–	–	–	–
13	Yes (No data on which medication)	–	–	–
14	Olanzapine (10)	Diazepam (10)/ Zopiklon (7.5)	–	Mirtazapin (30)
15	Risperidone (4)	Oxazepam (10)	–	Mirtazapin (30)
16	–	Zopiklon (7.5)	–	–
17	Olanzapine (20)	–	–	–
18	Olanzapine (20)	–	–	–
19	–	–	–	–
20	–	–	–	–
21	Olanzapine (15)	Zopiklon (7.5)	–	–
22	Risperidone (2)	–	–	–
23	–	Zolpidem (10)	–	–
24	Quetiapine (50)	–	–	Venlafaxin (300)
25	–	Nitrazepam (5)	–	–
26	Quetiapine (600)	–	–	–
27	–	–	–	–
28	–	Oxazepam (15)	–	–
29	–	–	–	–
30	Olanzapine (20)	–	Alimemazin (20)	–
31	–	–	–	Citalopram (20)
32	–	–	–	–
33	Risperidone (4)	–	–	–
34	–	–	–	–
35	Risperidone (4)	Oxazepam (10)/ Zopiklon (7.5)	–	–
36	Olanzapine (15)	–	–	–
37	–	–	–	–
38	–	–	–	–
39	–	Zopiklon (7.5)	–	–
40	–	–	–	–
41	–	–	–	–
42	Aripiprazole (15 mg/day); Flupentixol (2 mg/day)	–		Paroxetin (20)
43	Olanzapine (15)	–	–	–
44	Olanzapine (20)		Propiomazin (25)	–
45	Olanzapine (30)	–	–	–
46	Olanzapine (10)	–	–	–
47	–	Oxazepam (10)/ Zopiklon (7.5)	–	Paroxetin (20)

Supplementary Table S3. Fold change (FC) cutoffs distinguished 5% of metabolites between follow-up and baseline in healthy controls at FDR < 0.15.

Sample Type	Mode	No. of Metabolites FDR < 0.15		FC Cutoff for 5% Difference Allowance	FC Cutoff for Maximum FC
		Total	5%		
Serum	pC18	977	48.9	0.354	0.630
Serum	nC18	6	0.3	0.273	0.273
Serum	nHILIC	84	4.2	0.108	0.296
Serum	pHILIC	N/A	N/A	N/A	N/A
CSF	pC18	3	0.15	0.197	0.197
CSF	nC18	0	0	0.101	0.101
CSF	pHILIC	42	2.1	0.200	0.265
CSF	nHILIC	334	16.7	0.056	1.327

Supplementary Table S4. Number of metabolites after applying fold change (FC) cutoffs.

Type	Mode	FC Cutoff*	Follow-up vs. Baseline		FEP vs. Controls	
			Controls	FEP	Baseline	Follow-up
Serum	pC18	0.354	49	8	99	114
Serum	nC18	0.273	1	3	17	0
Serum	nHILIC	0.108	5	8	9	5
Serum	pHILIC	N/A	N/A	N/A	N/A	N/A
CSF	pC18	0.197	1	0	1	0
CSF	nC18	0.101	1	0	0	0
CSF	pHILIC	0.200	3	5	6	0
CSF	nHILIC	0.056	17	3	4	7

*Based on FC cutoffs distinguished 5% of metabolites between follow-up and baseline in healthy controls at FDR < 0.15. See Supplementary Table S3.

Supplementary Table S5. Clinical characteristics (mean ± SD) of FEP patients (n = 25) at baseline and 1.5-year follow-up.

	**Baseline**	**Follow-up**	***p*-value**
Psychiatric Assessments			
PANSS			
Positive	19.7 ± 6.2	11.7 ± 3.6*	<0.0001
Negative	17.5 ± 8.5	13.9 ± 6.0	0.0677
General	38.0 ± 11.6	28.8 ± 7.9*	0.0017
Total	75.2 ± 23.2	54.4 ± 14.2*	0.0002
Level of Functioning			
GAF Symptom	32.6 ± 8.7	59.2 ± 18.8*	<0.0001
GAF Function	39.8 ± 12.3	65.2 ± 17.0*	<0.0001
CGI Scores	4.5 ± 1.2	2.6 ± 1.5*	<0.0001
Cognitive Characteristics			
Speed of Processing			
TMT, Part A (sec)	35.7 ± 14.9	35.4 ± 24.2	0.9344
Fluency Test (# named)	21.6 ± 5.8	21.7 ± 6.8	0.9371
BACS-SC (# correct)	44.2 ± 11.8	47.5 ± 14.3*	0.0253
Attention/Vigilance			
CPT-IP (detectability)	2.1 ± 0.7	2.4 ± 0.6*	0.0048
Working Memory			
LNS (# correct trials)	12.8 ± 3.3	12.9 ± 2.9	0.8546
WMS-III SS (Sum forward and backward scores)	16.3 ± 3.2	15.5 ± 3.3	0.1131
Verbal Learning			
HVLT-R (Total number words recalled over 3 trials)	22.8 ± 5.4	22.0 ± 6.1	0.3517
Visual Learning			
NAB (3-trial total learning score)	18.4 ± 6.7	19.6 ± 5.6	0.1565
BVMT-R (3-trial total recall score)	21.8 ± 7.0	24.4 ± 6.7*	0.0169
Social Cognition			
MSCEIT-ME (branch score)	90.0 ± 11.7	90.6 ± 10.4	0.6634

*$p<0.05$ for paired t-test comparing baseline and follow-up data in the FEP group.
Abbreviations: PANSS, Positive and Negative Syndrome Scale Score; GAF, Global Assessment of Function; CGI, Clinical Global Impression; TMT, Trail Making Test; BACS-SC, Brief Assessment of Cognition in Schizophrenia-Symbol Coding Subtest; CPT-IP, Continuous Performance Test-Identical Pairs version; LNS, Letter Number Span test; WMS-III SS, Wechsler Memory Scale-3rd ed. Spatial Span subtest; HVLT-R, Hopkins Verbal Learning Test-Revised; NAB, Neuropsychological Assessment Bat.; BVMT-R, Brief Visuospatial Memory Test-Revised; MSCEIT-ME, Mayer-Salovey-Caruso Emotional Intelligence Test-Managing Emotions branch

Supplementary Table S6. CSF metabolites differed between FEP patients and healthy controls at baseline. Unidentified metabolites are highlighted in gray.

Mode	Metabolomic signatures	Log2 Fold Change (F_B/H_B)	Ave exp	*p*-value	FDR
HILIC+	Acanthicifoline	1.660	6.938	2.87×10^{-5}	2.82×10^{-2}
	Guvacoline	1.595	7.855	2.42×10^{-4}	4.76×10^{-2}
	Serotonin	1.592	7.694	5.54×10^{-4}	6.06×10^{-2}
	Pyriculol	1.270	8.038	3.03×10^{-3}	1.30×10^{-1}
	Athamantin	-0.542	9.639	5.17×10^{-4}	6.06×10^{-2}
	C6 H18 N2 O2	1.163	8.923	3.47×10^{-3}	1.36×10^{-1}
HILIC-	Citric acid	1.255	9.317	3.59×10^{-3}	1.11×10^{-1}
	Acetylenedicarboxylate	0.073	10.941	1.05×10^{-3}	5.67×10^{-2}
	Pyriculol	0.061	10.757	2.39×10^{-5}	7.78×10^{-3}
	Rhamnetin 3-(3''''-p-coumaryl-rhamnosyl)(1^{-3})-rhamnosyl-(1-6)-galactoside	-1.231	9.266	1.29×10^{-3}	6.00×10^{-2}
C18+	Serotonin	1.738	7.101	1.78×10^{-5}	1.77×10^{-2}
C18-	-	-	-	-	-

Supplementary Table S7. Serum metabolites differed between FEP patients and healthy controls at baseline. Unidentified metabolites are highlighted in gray.

Mode	Metabolomic signatures	Log2 Fold Change (F_B/H_B)	Ave exp	*p*-value	FDR
HILIC+	-	-	-	-	-
HILIC-	N-depyridomethyl-Indinavir	-0.478	9.751	$1.41x10^{-3}$	$8.15x10^{-2}$
	Norathyriol	-1.613	7.444	$2.12x10^{-4}$	$3.28x10^{-2}$
	C13 H13 N8 O3 S2	1.415	7.760	$2.01x10^{-3}$	$9.38x10^{-2}$
	C5 H5 N O4 S	0.144	10.153	$6.79x10^{-6}$	$3.14x10^{-3}$
	C11 H3 N O12 S2	-0.426	9.841	$4.59x10^{-3}$	$1.42x10^{-1}$
	C31 H44 N2 O5	-0.472	9.761	$1.38x10^{-3}$	$8.15x10^{-2}$
	C19 H3 N3 O7	-1.087	9.039	$2.90x10^{-3}$	$1.12x10^{-1}$
	C33 H56 N3 O7	-1.113	9.331	$2.65x10^{-3}$	$1.07x10^{-1}$
	C34 H25 N26	-1.977	7.615	$5.29x10^{-6}$	$3.14x10^{-3}$
C18+	Myxalamid A	1.134	7.911	$1.25x10^{-3}$	$5.88x10^{-3}$
	N-oleoyl methionine	0.951	7.811	$1.09x10^{-2}$	$2.86x10^{-2}$
	Val Val	0.802	8.498	$1.60x10^{-2}$	$3.75x10^{-2}$
	PE(20:5(5Z,8Z,11Z,14Z,17Z) /0:0)	0.758	9.016	$1.90x10^{-2}$	$4.29x10^{-2}$
	17-Methyl-18-norandrosta-4,13(17)-dien-3-one	0.737	8.013	$3.59x10^{-2}$	$7.00x10^{-2}$
	N-docosahexaenoyl GABA	0.653	8.899	$4.52x10^{-2}$	$8.41x10^{-2}$
	Trans-2, 3, 4-Trimethoxycinnamate	0.604	9.218	$2.79x10^{-2}$	$5.80x10^{-2}$
	S-Farnesyl Thioacetic Acid	0.594	8.532	$8.38x10^{-2}$	$1.36x10^{-1}$
	MG(0:0/18:0/0:0)	0.560	9.290	$5.31x10^{-2}$	$9.51x10^{-2}$
	Hexazinone	0.363	9.574	$7.24x10^{-2}$	$1.20x10^{-1}$
	estrone 3-sulfate	-0.402	9.366	$9.53x10^{-3}$	$2.63x10^{-2}$
	Nalorphine	-0.479	8.923	$9.30x10^{-2}$	$1.47x10^{-1}$
	Galalpha1-4Galbeta-Cer(d18:1/16:0)	-0.565	9.252	$6.72x10^{-2}$	$1.13x10^{-1}$
	Theobromine Esi+3.7969975	-0.613	8.394	$8.29x10^{-2}$	$1.35x10^{-1}$
	(3Z)-Phytochromobilin Esi+8.159999	-0.799	8.714	$2.30x10^{-2}$	$4.97x10^{-2}$
	Tetracaine	-0.998	7.046	$3.96x10^{-3}$	$1.35x10^{-2}$
	Serratanidine	-1.027	7.046	$1.16x10^{-3}$	$5.56x10^{-3}$
	N-acetyl-S-farnesyl-L-Cysteine	-1.042	7.069	$1.01x10^{-3}$	$5.18x10^{-3}$
	Iodoform	-1.062	7.075	$6.07x10^{-4}$	$4.12x10^{-3}$
	Pseudopelletierine	-1.079	7.210	$7.25x10^{-4}$	$4.54x10^{-3}$
	Asp Lys Lys	-1.094	7.016	$8.69x10^{-4}$	$4.86x10^{-3}$
	Lysyl-Tyrosyl-Lysine	-1.106	7.136	$5.34x10^{-4}$	$3.84x10^{-3}$
	Pseudoargiopinin III	-1.132	6.952	$4.86x10^{-4}$	$3.73x10^{-3}$
	Arg Gln Ile	-1.139	7.080	$4.86x10^{-4}$	$3.73x10^{-3}$
	Thr Lys Lys Esi+5.067995	-1.161	7.027	$2.85x10^{-4}$	$3.09x10^{-3}$
	Cadiamine	-1.172	7.177	$4.87x10^{-4}$	$3.73x10^{-3}$
	Gabapentin	-1.236	7.060	$1.96x10^{-4}$	$2.40x10^{-3}$
	C25 H28 N9 O2 S	1.117	6.695	$6.75x10^{-5}$	$1.29x10^{-3}$
	C24 H49 N8 O6	0.987	8.638	$1.58x10^{-2}$	$3.73x10^{-2}$
	C19 H39 N8 O4	0.960	8.783	$5.08x10^{-2}$	$9.20x10^{-2}$
	C16 H33 N8 O3	0.946	8.793	$5.88x10^{-2}$	$1.02x10^{-1}$
	C21 H39 N15	0.877	8.800	$5.59x10^{-2}$	$9.90x10^{-2}$
	<none> Esi+0.8249996	0.847	7.573	$1.60x10^{-2}$	$3.75x10^{-2}$
	<none> Esi+4.9589953	0.796	9.066	$3.96x10^{-2}$	$7.51x10^{-2}$

C15 H28 N7 O2	0.767	8.852	8.40x10^{-2}	1.36x10^{-1}
C11 H19 N O2	-0.436	9.011	8.50x10^{-2}	1.37x10^{-1}
C31 H57 O9 S	-0.531	8.916	6.51x10^{-2}	1.10x10^{-1}
C28 H41 N8 O3 S	-0.648	7.228	6.31x10^{-2}	1.08x10^{-1}
C17 H29 N O4	-0.732	8.490	2.59x10^{-2}	5.47x10^{-2}
C36 H59 N2 O10 S	-0.993	7.036	1.25x10^{-3}	5.88x10^{-3}
C13 H26 O6	-1.012	7.058	1.33x10^{-3}	6.12x10^{-3}
C34 H45 N13 S	-1.020	7.082	7.39x10^{-4}	4.56x10^{-3}
<none> Esi+6.316997	-1.026	7.163	7.22x10^{-4}	4.54x10^{-3}
C35 H73 N O17	-1.034	7.072	1.32x10^{-3}	6.12x10^{-3}
C21 H35 N O4	-1.035	8.788	1.05x10^{-4}	1.60x10^{-3}
C9 H28 N11 O4	-1.041	6.976	1.60x10^{-3}	6.89x10^{-3}
<none> Esi+6.248999	-1.047	7.065	9.64x10^{-4}	5.16x10^{-3}
C20 H41 N8 O5	-1.057	7.109	8.10x10^{-4}	4.74x10^{-3}
<none> Esi+6.096992	-1.061	7.123	7.81x10^{-4}	4.66x10^{-3}
<none> Esi+6.098001	-1.063	7.033	9.37x10^{-4}	5.08x10^{-3}
C21 H35 N3 O4	-1.068	7.266	2.20x10^{-3}	8.83x10^{-3}
C33 H53 N5 O6 Esi+5.3609943	-1.074	7.130	1.00x10^{-3}	5.18x10^{-3}
<none> Esi+5.583006	-1.076	7.213	8.50x10^{-4}	4.85x10^{-3}
C23 H49 N O11	-1.077	7.118	1.59x10^{-3}	6.86x10^{-3}
C16 H31 N20	-1.077	7.075	6.57x10^{-4}	4.29x10^{-3}
C28 H57 N8 O9	-1.080	7.101	6.82x10^{-4}	4.39x10^{-3}
C19 H31 N5 O4	-1.081	7.115	5.33x10^{-4}	3.84x10^{-3}
C18 H35 N20 O	-1.083	6.959	5.81x10^{-4}	4.03x10^{-3}
C33 H69 N O16	-1.086	7.101	8.76x10^{-4}	4.88x10^{-3}
C17 H37 N O8	-1.088	7.211	5.54x10^{-4}	3.89x10^{-3}
C21 H35 N3 O4 Esi+5.0669937	-1.088	7.153	2.86x10^{-3}	1.09x10^{-2}
C26 H41 N2 O S	-1.089	7.060	7.57x10^{-4}	4.60x10^{-3}
C23 H33 N2 O5	-1.092	7.116	5.55x10^{-4}	3.89x10^{-3}
C27 H49 N12 O4	-1.092	7.175	4.51x10^{-4}	3.73x10^{-3}
<none> Esi+6.174994	-1.096	7.036	6.25x10^{-4}	4.17x10^{-3}
C36 H71 N8 O13	-1.097	7.046	4.79x10^{-4}	3.73x10^{-3}
C17 H35 N15 S	-1.098	7.041	7.75x10^{-4}	4.66x10^{-3}
C16 H33 N8 O3 Esi+6.3790035	-1.099	6.945	7.56x10^{-4}	4.60x10^{-3}
C23 H35 N10	-1.103	7.060	1.79x10^{-4}	2.33x10^{-3}
C36 H71 N5 O13 S	-1.104	7.101	4.13x10^{-4}	3.58x10^{-3}
<none> Esi+6.383996	-1.108	7.025	3.89x10^{-4}	3.58x10^{-3}
C19 H38 N3 O7	-1.112	7.023	9.20x10^{-4}	5.05x10^{-3}
C11 H32 N11 O5	-1.113	7.101	4.67x10^{-4}	3.73x10^{-3}
C15 H30 O7	-1.114	7.074	5.38x10^{-4}	3.84x10^{-3}
C17 H34 N3 O6	-1.116	7.027	6.75x10^{-4}	4.37x10^{-3}
C13 H36 N11 O6	-1.117	7.071	4.60x10^{-4}	3.73x10^{-3}
C27 H45 N2 O8	-1.122	6.994	4.41x10^{-4}	3.73x10^{-3}
C30 H61 N8 O10 S	-1.125	7.210	4.11x10^{-4}	3.58x10^{-3}
C17 H2 N2 O18 S2	-1.126	7.159	8.37x10^{-4}	4.85x10^{-3}
C18 H37 N8 O4 S	-1.127	6.961	1.37x10^{-3}	6.30x10^{-3}
C33 H61 N12 O7 S	-1.129	7.092	4.86x10^{-4}	3.73x10^{-3}
C22 H45 N8 O6	-1.139	7.111	3.10x10^{-4}	3.19x10^{-3}
C29 H53 N12 O5 S	-1.166	7.097	5.87x10^{-4}	4.04x10^{-3}
C29 H55 N15 O5	-1.166	6.943	5.15x10^{-4}	3.80x10^{-3}
C30 H59 N8 O10	-1.168	7.074	4.88x10^{-4}	3.73x10^{-3}
C27 H57 N O13	-1.172	7.074	6.55x10^{-4}	4.29x10^{-3}
C33 H53 N5 O6	-1.172	7.037	1.02x10^{-3}	5.18x10^{-3}

	C28 H55 N5 O9 S	-1.173	7.039	1.16x10^{-3}	5.56x10^{-3}
	C23 H32 N11	-1.175	6.780	7.94x10^{-5}	1.38x10^{-3}
	C19 H41 N O9	-1.179	7.091	4.02x10^{-4}	3.58x10^{-3}
	C23 H N2 O21	-1.18	7.128	6.07x10^{-4}	4.12x10^{-3}
	C24 H49 N8 O7	-1.188	6.991	3.69x10^{-4}	3.53x10^{-3}
	C8 Cl3 N3 O S5	-1.191	7.067	5.16x10^{-4}	3.80x10^{-3}
	C20 H33 N3 O S	-1.194	7.081	2.97x10^{-4}	3.15x10^{-3}
	C12 H29 N9 O4	-1.203	7.089	4.25x10^{-4}	3.61x10^{-3}
	C29 H61 N O14	-1.208	7.062	4.11x10^{-4}	3.58x10^{-3}
	C25 H53 N O12	-1.226	7.016	5.39x10^{-4}	3.84x10^{-3}
	C21 H45 N O10	-1.276	7.020	2.23x10^{-4}	2.56x10^{-3}
	C15 H33 N13 O4	-1.296	7.137	9.40x10^{-5}	1.50x10^{-3}
C18-	2-Thiopheneacrylic acid	0.560	10.133	3.17x10^{-3}	1.29x10^{-1}
	C27 H45 O7	-0.852	9.650	3.00x10^{-3}	1.29x10^{-1}
	PS(13:0/12:0)	-0.870	9.685	3.36x10^{-3}	1.29x10^{-1}
	PS(13:0/12:0) Esi-12.402006	-0.960	9.619	1.48x10^{-3}	1.29x10^{-1}
	10-hydroxy-2E,8E-Decadiene-4,6-diynoic acid	-1.510	8.506	3.69x10^{-4}	6.54x10^{-2}
	<none> Esi-4.960006	0.946	9.732	6.87x10^{-4}	9.66x10^{-2}
	C22 H19 N8 O	0.390	10.221	3.15x10^{-3}	1.29x10^{-1}
	C41 H69 N3 O12	-0.806	9.670	4.05x10^{-3}	1.42x10^{-1}
	C26 H37 N6 O7 Esi-10.69201	-0.824	9.429	4.42x10^{-3}	1.43x10^{-1}
	C22 H49 N17 O4 S	-0.871	9.583	3.21x10^{-3}	1.29x10^{-1}
	C28 H55 N19 O2 S	-0.874	9.473	4.93x10^{-3}	1.48x10^{-1}
	C32 H63 N6 O9 S	-0.887	9.504	2.96x10^{-3}	1.29x10^{-1}
	C33 H55 N3 O9	-0.949	9.633	1.79x10^{-3}	1.29x10^{-1}
	C37 H55 O13	-1.168	9.288	3.88x10^{-4}	6.54x10^{-2}
	C31 H19 N4 O30 S	-1.183	8.539	2.56x10^{-3}	1.29x10^{-1}
	C10 H12 O6 S	-1.772	8.340	1.59x10^{-5}	6.69x10^{-3}
	C19 H18 O10 S	-1.988	7.983	5.39x10^{-7}	4.55x10^{-4}

Supplementary Table S8. CSF metabolites differed between FEP patients and healthy controls at follow-up. The fold changes of CSF metabolites with statistically significant between-group difference at baseline are shown. Unidentified metabolites are highlighted in gray.

Mode	Metabolomic signatures	Log2 Fold Change (F_{FU}/H_{FU})	Ave exp	*p*-value	FDR	Log2 Fold Change (F_B/H_B)
HILIC+	-	-	-	-	-	
HILIC-	Citric acid	1.267	8.934	$4.99x10^{-3}$	$1.12x10^{-1}$	1.255
	Phenolphthalin	1.145	9.555	$3.26x10^{-3}$	$1.05x10^{-1}$	
	2,4-Diamino-6,7-dimethoxyquinazoline	1.100	9.632	$4.39x10^{-3}$	$1.12x10^{-1}$	
	Acetylenedicarboxylate	0.069	10.938	$4.13x10^{-3}$	$1.12x10^{-1}$	0.073
	C6 H12 O8 S	-0.481	10.393	$6.38x10^{-3}$	$1.34x10^{-1}$	
	C30 H7 N4 O2	-0.799	9.858	$7.36x10^{-3}$	$1.45x10^{-1}$	
	C41 H9 N2	-1.101	9.533	$1.43x10^{-3}$	$6.27x10^{-2}$	
C18+	-	-	-	-	-	
C18-	-	-	-	-	-	

Supplementary Table S9. Serum metabolites differed between patients and healthy controls at follow-up. The fold changes of serum metabolites with statistically significant between-group difference at baseline are shown. Unidentified metabolites are highlighted in gray.

Mode	Metabolomic signatures	Log2 Fold Change (F_{FU}/H_{FU})	Ave exp	*p*-value	FDR	Log2 Fold Change (F_B/H_B)
HILIC+	-	-	-	-	-	
HILIC-	AG-041R	0.469	9.793	$1.15x10^{-3}$	$5.33x10^{-2}$	
	Urocanic acid	-0.629	9.669	$2.08x10^{-3}$	$7.42x10^{-2}$	
	Norathyriol	-1.863	8.116	$2.44x10^{-5}$	$1.13x10^{-2}$	-1.613
	C33 H34 N20 O3	-0.366	9.822	$4.73x10^{-3}$	$1.41x10^{-1}$	
	C25 H12 N O7	-1.292	7.443	$2.27x10^{-3}$	$7.80x10^{-2}$	
C18+	N-oleoyl methionine	1.118	7.649	$5.09x10^{-3}$	$1.09x10^{-2}$	0.951
	S-Farnesyl Thioacetic Acid	1.052	8.086	$4.78x10^{-3}$	$1.04x10^{-2}$	0.594
	Bis (2-hydroxypropyl) amine Esi+0.6719987	0.658	9.207	$5.22x10^{-2}$	$8.09x10^{-2}$	
	Bis (2-hydroxypropyl) amine Esi+0.6719987	0.658	9.207	$5.22x10^{-2}$	$8.09x10^{-2}$	
	Poloxalene	0.362	9.532	$3.33x10^{-3}$	$7.65x10^{-3}$	
	Isobutyrylglycine methyl ester	-0.392	9.571	$2.40x10^{-2}$	$4.19x10^{-2}$	
	Mesuagin	-0.400	9.283	$3.30x10^{-3}$	$7.61x10^{-3}$	
	PC(20:2(11Z,14Z)/0:0)	-0.457	9.520	$6.89x10^{-3}$	$1.42x10^{-2}$	
	Lys Met Lys	-0.484	9.054	$3.71x10^{-2}$	$6.12x10^{-2}$	
	1,3-Dimethyl-6,8-isoquinolinediol	-0.56	8.276	$1.00x10^{-1}$	$1.42x10^{-1}$	
	Theophylline	-0.58	9.512	$3.84x10^{-3}$	$8.53x10^{-3}$	
	Theobromine Esi+3.7969975	-0.612	8.292	$8.48x10^{-2}$	$1.23x10^{-1}$	-0.613
	Hypoxanthine	-0.660	8.949	$8.81x10^{-2}$	$1.27x10^{-1}$	
	Kurilensoside G	-0.693	8.877	$1.12x10^{-2}$	$2.17x10^{-2}$	
	Myxalamid A	-0.721	7.524	$5.15x10^{-2}$	$8.03x10^{-2}$	1.134
	estrone 3-sulfate	-0.731	9.166	$1.14x10^{-4}$	$4.88x10^{-4}$	-0.402
	Asp Lys Lys	-1.385	7.885	$7.55x10^{-5}$	$3.52x10^{-4}$	-1.094
	Tetracaine	-1.432	7.943	$1.26x10^{-4}$	$5.20x10^{-4}$	-0.998
	N-acetyl-S-farnesyl-L-Cysteine	-1.507	7.859	$2.38x10^{-5}$	$1.50x10^{-4}$	-1.042
	Pseudopelletierine	-1.524	7.909	$6.68x10^{-5}$	$3.16x10^{-4}$	-1.079
	Gabapentin	-1.541	7.891	$3.53x10^{-5}$	$1.95x10^{-4}$	-1.236
	Serratanidine	-1.547	7.767	$1.78x10^{-5}$	$1.21x10^{-4}$	-1.027
	Lysyl-Tyrosyl-Lysine	-1.548	7.908	$2.61x10^{-5}$	$1.60x10^{-4}$	-1.106
	Iodoform	-1.556	7.795	$1.53x10^{-5}$	$1.15x10^{-4}$	-1.062
	Thr Lys Lys Esi+5.067995	-1.583	7.816	$1.23x10^{-5}$	$1.02x10^{-4}$	-1.161
	Arg Gln Ile	-1.590	7.918	$9.69x10^{-6}$	$9.02x10^{-5}$	-1.139
	Pseudoargiopinin III	-1.618	7.755	$5.56x10^{-6}$	$6.41x10^{-5}$	-1.132
	Cadiamine	-1.687	7.975	$1.65x10^{-5}$	$1.18x10^{-4}$	-1.172
	C16 H30 N7 O2	1.795	8.411	$4.27x10^{-5}$	$2.22x10^{-4}$	
	C20 H49 N17 O4	1.744	7.951	$9.77x10^{-5}$	$4.30x10^{-4}$	
	C19 H39 N8 O4	1.627	7.982	$8.83x10^{-5}$	$3.97x10^{-4}$	0.960
	C21 H39 N15	1.588	8.018	$8.93x10^{-5}$	$4.00x10^{-4}$	0.877
	C20 H41 N8 O4	1.576	8.050	$1.21x10^{-4}$	$5.11x10^{-4}$	
	C16 H33 N8 O3	1.524	8.074	$1.73x10^{-4}$	$6.59x10^{-4}$	0.946
	C24 H49 N8 O6	1.475	7.866	$3.61x10^{-4}$	$1.24x10^{-3}$	0.987
	C17 H32 N7 O3	1.415	8.190	$6.38x10^{-4}$	$1.95x10^{-3}$	
	C15 H28 N7 O2	1.303	8.145	$1.51x10^{-3}$	$3.98x10^{-3}$	0.767
	C13 H24 N7 O2 Esi+5.043013	1.156	8.349	$2.40x10^{-3}$	$5.89x10^{-3}$	
	C13 H24 N7 O2	1.121	8.282	$3.70x10^{-3}$	$8.27x10^{-3}$	
	C12 H22 N7 O	1.036	8.893	$7.86x10^{-2}$	$1.16x10^{-1}$	

C25 H28 N9 O2 S	0.952	6.592	7.05x10^{-4}	2.13x10^{-3}	1.117
C21 H26 N24 O3	0.511	8.678	1.03x10^{-1}	1.45x10^{-1}	
<none> Esi+4.914001	0.495	9.189	2.90x10^{-2}	4.93x10^{-2}	
C23 H37 N8	0.491	8.895	6.45x10^{-2}	9.73x10^{-2}	
<none> Esi+4.9589953	0.379	9.419	4.82x10^{-2}	7.61x10^{-2}	0.796
C16 H31 N O4	-0.371	9.568	4.44x10^{-3}	9.77x10^{-3}	
C7 H4 O	-0.389	9.331	2.60x10^{-2}	4.49x10^{-2}	
C25 H45 N O6	-0.518	8.753	8.47x10^{-2}	1.23x10^{-1}	
C13 H32 N12 O4 S	-0.530	9.124	4.05x10^{-2}	6.56x10^{-2}	
<none> Esi+6.1630077	-0.575	8.566	7.98x10^{-2}	1.17x10^{-1}	
<none> Esi+5.929998	-0.830	8.872	3.31x10^{-3}	7.61x10^{-3}	
<none> Esi+6.231005	-0.885	8.621	3.36x10^{-3}	7.69x10^{-3}	
C26 H39 N O10	-1.126	7.875	2.53x10^{-3}	6.13x10^{-3}	
<none> Esi+5.7470055	-1.215	8.307	8.12x10^{-4}	2.38x10^{-3}	
C22 H45 N8 O6 S	-1.245	8.186	7.97x10^{-4}	2.35x10^{-3}	
<none> Esi+6.091003	-1.253	8.687	2.47x10^{-5}	1.54x10^{-4}	
C16 H33 N8 O3 Esi+6.3790035	-1.378	7.823	1.30x10^{-4}	5.32x10^{-4}	-1.099
<none> Esi+6.383996	-1.437	7.811	1.44x10^{-4}	5.74x10^{-4}	-1.108
<none> Esi+6.316997	-1.456	7.832	6.49x10^{-5}	3.08x10^{-4}	-1.026
C34 H45 N13 S	-1.474	7.859	1.29x10^{-5}	1.03x10^{-4}	-1.020
C36 H59 N2 O10 S	-1.484	7.791	2.28x10^{-5}	1.46x10^{-4}	-0.993
C20 H41 N8 O5	-1.485	7.890	2.40x10^{-5}	1.50x10^{-4}	-1.057
C19 H31 N5 O4	-1.497	7.868	3.05x10^{-5}	1.76x10^{-4}	-1.081
C16 H31 N20	-1.499	7.864	2.65x10^{-5}	1.61x10^{-4}	-1.077
C27 H49 N12 O4	-1.503	7.881	3.61x10^{-5}	1.97x10^{-4}	-1.092
C18 H35 N20 O	-1.509	7.782	1.21x10^{-5}	1.01x10^{-4}	-1.083
C13 H26 O6	-1.510	7.831	2.71x10^{-5}	1.63x10^{-4}	-1.012
<none> Esi+6.174994	-1.511	7.807	4.69x10^{-5}	2.41x10^{-4}	-1.096
C23 H35 N10	-1.514	7.819	7.37x10^{-6}	7.40x10^{-5}	-1.103
C15 H33 N13 O4	-1.535	7.844	5.30x10^{-5}	2.70x10^{-4}	-1.296
C23 H32 N11	-1.539	7.790	4.59x10^{-6}	5.57x10^{-5}	-1.175
C36 H71 N5 O13 S	-1.540	7.860	1.64x10^{-5}	1.18x10^{-4}	-1.104
C11 H32 N11 O5	-1.543	7.897	2.65x10^{-5}	1.61x10^{-4}	-1.113
<none> Esi+6.096992	-1.544	7.925	1.18x10^{-5}	9.96x10^{-5}	-1.061
C22 H45 N8 O6	-1.548	7.866	1.69x10^{-5}	1.19x10^{-4}	-1.139
<none> Esi+6.098001	-1.550	7.818	1.80x10^{-5}	1.21x10^{-4}	-1.063
C28 H57 N8 O9	-1.552	7.857	1.65x10^{-5}	1.18x10^{-4}	-1.080
<none> Esi+6.248999	-1.558	7.831	1.59x10^{-5}	1.17x10^{-4}	-1.047
C9 H28 N11 O4	-1.558	7.815	1.93x10^{-5}	1.27x10^{-4}	-1.041
C23 H33 N2 O5	-1.563	7.860	1.82x10^{-5}	1.21x10^{-4}	-1.092
C21 H35 N3 O4 Esi+5.0669937	-1.577	8.018	4.14x10^{-5}	2.17x10^{-4}	-1.088
C36 H71 N8 O13	-1.582	7.795	1.70x10^{-5}	1.19x10^{-4}	-1.097
C21 H35 N3 O4	-1.582	8.060	3.86x10^{-5}	2.07x10^{-4}	-1.068
C24 H49 N8 O7	-1.592	7.824	2.07x10^{-5}	1.35x10^{-4}	-1.188
C15 H30 O7	-1.594	7.872	1.63x10^{-5}	1.18x10^{-4}	-1.114
C17 H35 N15 S	-1.595	7.851	1.27x10^{-5}	1.03x10^{-4}	-1.098
C13 H36 N11 O6	-1.596	7.854	1.37x10^{-5}	1.08x10^{-4}	-1.117
C29 H55 N15 O5	-1.616	7.727	1.69x10^{-5}	1.19x10^{-4}	-1.166
C18 H37 N8 O4 S	-1.619	7.887	2.35x10^{-5}	1.49x10^{-4}	-1.127
C35 H73 N O17	-1.621	7.871	9.95x10^{-6}	9.15x10^{-5}	-1.034
C33 H69 N O16	-1.622	7.850	3.63x10^{-5}	1.97x10^{-4}	-1.086
C19 H41 N O9	-1.626	7.945	1.77x10^{-5}	1.21x10^{-4}	-1.179
C26 H41 N2 O S	-1.639	7.804	5.16x10^{-6}	6.15x10^{-5}	-1.089
C27 H45 N2 O8	-1.640	7.707	1.18x10^{-5}	9.96x10^{-5}	-1.122
C19 H38 N3 O7	-1.640	7.835	1.28x10^{-5}	1.03x10^{-4}	-1.112
C33 H61 N12 O7 S	-1.643	7.856	1.12x10^{-5}	9.70x10^{-5}	-1.129

	<none> Esi+5.583006	-1.645	7.980	9.98x10^{-6}	9.15x10^{-5}	-1.076
	C17 H37 N O8	-1.660	7.959	5.74x10^{-6}	6.48x10^{-5}	-1.088
	C17 H34 N3 O6	-1.661	7.776	1.78x10^{-5}	1.21x10^{-4}	-1.116
	C30 H61 N8 O10 S	-1.677	7.948	5.58x10^{-6}	6.41x10^{-5}	-1.125
	C17 H2 N2 O18 S2	-1.683	7.985	1.19x10^{-5}	9.99x10^{-5}	-1.126
	C29 H53 N12 O5 S	-1.684	7.927	1.16x10^{-5}	9.96x10^{-5}	-1.166
	C12 H29 N9 O4	-1.684	7.828	5.56x10^{-5}	2.78x10^{-4}	-1.203
	C20 H33 N3 O S	-1.689	7.853	1.08x10^{-5}	9.61x10^{-5}	-1.194
	C33 H53 N5 O6 Esi+5.3609943	-1.706	7.858	8.63x10^{-6}	8.34x10^{-5}	-1.074
	C23 H49 N O11	-1.712	7.973	6.49x10^{-6}	6.89x10^{-5}	-1.077
	C30 H59 N8 O10	-1.730	7.897	6.97x10^{-6}	7.20x10^{-5}	-1.168
	C23 H N2 O21	-1.738	7.946	1.46x10^{-5}	1.11x10^{-4}	-1.180
	C25 H53 N O12	-1.743	7.966	7.00x10^{-6}	7.20x10^{-5}	-1.226
	C33 H53 N5 O6	-1.746	7.861	1.41x10^{-5}	1.08x10^{-4}	-1.172
	C28 H55 N5 O9 S	-1.755	7.847	1.36x10^{-5}	1.08x10^{-4}	-1.173
	C8 Cl3 N3 O S5	-1.781	7.860	7.67x10^{-6}	7.64x10^{-5}	-1.191
	C21 H45 N O10	-1.781	7.885	7.27x10^{-6}	7.36x10^{-5}	-1.276
	C27 H57 N O13	-1.789	7.861	1.10x10^{-5}	9.65x10^{-5}	-1.172
	C29 H61 N O14	-1.799	7.843	9.23x10^{-6}	8.85x10^{-5}	-1.208
C18-	-	-	-	-	-	

Supplementary Table S10. CSF metabolites differed between follow-up and baseline in FEP patients. Unidentified metabolites are highlighted in gray.

Mode	Metabolomic signatures	Log2 Fold Change (F_{FU}/F_{B})	Ave exp	*p*-value	FDR
HILIC+	Deoxymiroestrol	0.220	9.823	1.30×10^{-11}	6.38×10^{-9}
	Trans-3-Aminocyclopentane-1-carboxylic acid	-0.556	8.558	6.25×10^{-3}	6.40×10^{-2}
	C18 H30 N2 O4	-0.563	7.978	1.35×10^{-2}	9.86×10^{-2}
	C12 H20 N2	-0.623	7.542	2.13×10^{-2}	1.27×10^{-1}
	C20 H43 N4 O2	-1.302	7.557	6.78×10^{-4}	1.48×10^{-2}
HILIC-	Lasiocarpine	-0.062	10.542	4.34×10^{-13}	2.83×10^{-10}
	C20 H5 N3 O7	0.900	7.614	8.87×10^{-3}	1.20×10^{-1}
	<none> Esi-1.6579995	-0.067	9.627	6.69×10^{-3}	1.06×10^{-1}
C18+	-	-	-	-	-
C18-	-	-	-	-	-

Supplementary Table S11. Serum metabolites differed between follow-up and baseline in FEP patients. Unidentified metabolites are highlighted in gray.

Mode	Metabolomic signatures	Log2 Fold Change (F_{FU}/F_B)	Ave exp	*p*-value	FDR
HILIC+	-	-	-	-	-
HILIC-	15(S)-15-methyl PGF2α isopropyl ester	0.579	8.209	$9.65X10^{-3}$	$6.72X10^{-2}$
	13-Eicosenoic acid	0.540	7.805	$2.07X10^{-2}$	$1.02X10^{-1}$
	Nocodazole	-0.300	7.864	$3.83X10^{-2}$	$1.46X10^{-1}$
	Taurochenodeoxycholic acid	-0.645	8.567	$1.76X10^{-2}$	$9.19X10^{-2}$
	C34 H25 N26	1.003	7.401	$1.72X10^{-4}$	$5.11X10^{-3}$
	C20 H2 N2 O2	0.776	7.886	$9.94X10^{-3}$	$6.77X10^{-2}$
	C7 H5 N O S	0.492	8.365	$3.14x10^{-30}$	$2.90x10^{-27}$
	C25 H12 N O7	-0.332	6.708	$2.58x10^{-19}$	$1.19x10^{-16}$
C18+	Asp Lys Lys	0.398	6.890	$4.69X10^{-3}$	$1.96X10^{-2}$
	N-methylundec-10-enamide	0.354	8.978	$7.68X10^{-3}$	$2.62X10^{-2}$
	Myxalamid A	-0.807	7.812	$7.48X10^{-4}$	$5.93X10^{-3}$
	C23 H32 N11	0.680	6.671	$6.53X10^{-5}$	$1.32X10^{-3}$
	<none> Esi+6.1630077	0.434	7.894	$2.34X10^{-2}$	$5.81X10^{-2}$
	C16 H33 N8 O3	0.417	6.826	$2.17X10^{-3}$	$1.16X10^{-2}$
	C21 H35 N O4	0.399	8.784	$1.16X10^{-2}$	$3.45X10^{-2}$
	C18 H35 N20 O	0.361	6.786	$1.64X10^{-3}$	$1.02X10^{-2}$
C18-	C10 H12 O6 S	0.942	8.249	$3.84X10^{-5}$	$8.09X10^{-3}$
	C8 H8 O6 S	0.324	8.825	$1.02X10^{-3}$	$7.83X10^{-2}$
	C19 H18 O10 S	-0.296	7.269	$3.64X10^{-6}$	$1.53X10^{-3}$

Supplementary Table S12. Follow-up-associated CSF metabolites in FEP patients. Unidentified metabolites are highlighted in gray.

FEP_{FU}/FEP_{B}- HC_{FU}/ HC_{B}

Mode	Metabolomic signatures	Log2 Fold Change (F_{FU}/F_{B})	Ave exp	*p*-value
HILIC+	Deoxymiroestrol	0.220	$1.30x10^{-11}$	$6.38x10^{-9}$
	Trans-3-Aminocyclopentane-1-carboxylic acid	-0.556	$6.25x10^{-3}$	$6.40x10^{-2}$
	C18 H30 N2 O4	-0.563	$1.35x10^{-2}$	$9.86x10^{-2}$
	C12 H20 N2	-0.623	$2.13x10^{-2}$	$1.27x10^{-1}$
	C20 H43 N4 O2	-1.302	$6.78x10^{-4}$	$1.48x10^{-2}$
HILIC-	C20 H5 N3 O7	0.900	$8.87x10^{-3}$	$1.20x10^{-1}$
	Lasiocarpine	-0.062	$4.34x10^{-13}$	$2.83x10^{-10}$
	<none> Esi-1.6579995	-0.067	$6.69x10^{-3}$	$1.06x10^{-1}$
C18+	-	-	-	-
C18-	-	-	-	-

FEP_{B}/HC_{B} (≠FEP_{FU}/HC_{FU} or =- FEP_{FU}/HC_{FU})

Mode	Metabolomic signatures	Log2 Fold Change (F_{B}/ H_{B})	Ave exp	*p*-value
HILIC+	Acanthicifoline	1.660	$2.87x10^{-5}$	$2.82x10^{-2}$
	Guvacoline	1.595	$2.42x10^{-4}$	$4.76x10^{-2}$
	Serotonin	1.592	$5.54x10^{-4}$	$6.06x10^{-2}$
	Pyriculol	1.270	$3.03x10^{-3}$	$1.30x10^{-1}$
	Athamantin	-0.542	$5.17x10^{-4}$	$6.06x10^{-2}$
	C6 H18 N2 O2	1.163	$3.47x10^{-3}$	$1.36x10^{-1}$
HILIC-	Pyriculol	0.061	$2.39x10^{-5}$	$7.78x10^{-3}$
	Rhamnetin 3-(3''''-p-coumaryl-rhamnosyl)(1-3)-rhamnosyl-(1-6)-galactoside	-1.231	$1.29x10^{-3}$	$6.00x10^{-2}$
C18+	Serotonin	1.738	$1.78x10^{-5}$	$1.77x10^{-2}$
C18-	-	-	-	-

FEP_{FU}/HC_{FU} (≠FEP_{B}/HC_{B} or =- FEP_{B}/HC_{B})

Mode	Metabolomic signatures	Log2 Fold Change (F_{FU}/ H_{FU})	Ave exp	*p*-value
HILIC+	-	-	-	-
HILIC-	Phenolphthalin	1.145	$3.26x10^{-3}$	$1.05x10^{-1}$
	2,4-Diamino-6,7-dimethoxyquinazoline	1.100	$4.39x10^{-3}$	$1.12x10^{-1}$
	C6 H12 O8 S	-0.481	$6.38x10^{-3}$	$1.34x10^{-1}$
	C30 H7 N4 O2	-0.799	$7.36x10^{-3}$	$1.45x10^{-1}$
	C41 H9 N2	-1.101	$1.43x10^{-3}$	$6.27x10^{-2}$
C18+	-	-	-	-
C18-	-	-	-	-

Supplementary Table S13. Follow-up-associated serum metabolites in FEP patients. Unidentified metabolites are highlighted in gray.

FEP_{FU}/FEP_{B}- HC_{FU}/ HC_{B}

Mode	Metabolomic signatures	Log2 Fold Change (F_{FU}/F_{B})	Ave exp	*p*-value
HILIC+	-	-	-	-
HILIC-	15(S)-15-methyl PGF2α isopropyl ester	0.579	$9.65x10^{-3}$	$6.72x10^{-2}$
	13-eicosenoic acid	0.540	$2.07x10^{-2}$	$1.02x10^{-1}$
	Nocodazole	-0.300	$3.83x10^{-2}$	$1.46x10^{-1}$
	Taurochenodeoxycholic acid Esi-3.2800019	-0.645	$1.76x10^{-2}$	$9.19x10^{-2}$
	C34 H25 N26	1.003	$1.72x10^{-4}$	$5.11x10^{-3}$
	C20 H2 N2 O2	0.776	$9.94x10^{-3}$	$6.77x10^{-2}$
	C7 H5 N O S	0.492	$3.14x10^{-30}$	$2.90x10^{-27}$
C18+	N-methylundec-10-enamide Esi+8.5699835	0.354	$7.68x10^{-3}$	$2.62x10^{-2}$
	Myxalamid A	-0.807	$7.48x10^{-4}$	$5.93x10^{-3}$
	C21 H35 N O4	0.399	$1.16x10^{-2}$	$3.45x10^{-2}$
C18-	C10 H12 O6 S	0.942	$3.84x10^{-5}$	$8.09x10^{-3}$
	C8 H8 O6 S	0.324	$1.02x10^{-3}$	$7.83x10^{-2}$

FEP_{B}/HC_{B} (≠FEP_{FU}/HC_{FU} or =- FEP_{FU}/HC_{FU})

Mode	Metabolomic signatures	Log2 Fold Change (F_{B}/ H_{B})	Ave exp	*p*-value
HILIC+	-	-	-	-
HILIC-	N-depyridomethyl-Indinavir	-0.478	$1.41x10^{-3}$	$8.15x10^{-2}$
	C13 H13 N8 O3 S2	1.415	$2.01x10^{-3}$	$9.38x10^{-2}$
	C5 H5 N O4 S	0.144	$6.79x10^{-6}$	$3.14x10^{-3}$
	C11 H3 N O12 S2	-0.426	$4.59x10^{-3}$	$1.42x10^{-1}$
	C31 H44 N2 O5	-0.472	$1.38x10^{-3}$	$8.15x10^{-2}$
	C19 H3 N3 O7	-1.087	$2.90x10^{-3}$	$1.12x10^{-1}$
	C33 H56 N3 O7	-1.113	$2.65x10^{-3}$	$1.07x10^{-1}$
	C34 H25 N26	-1.977	$5.29x10^{-6}$	$3.14x10^{-3}$
C18+	Myxalamid A	1.134	$1.25x10^{-3}$	$5.88x10^{-3}$
	Val Val	0.802	$1.60x10^{-2}$	$3.75x10^{-2}$
	PE(20:5(5Z,8Z,11Z,14Z,17Z)/0:0)	0.758	$1.90x10^{-2}$	$4.29x10^{-2}$
	17-Methyl-18-norandrosta-4,13(17)-dien-3-one	0.737	$3.59x10^{-2}$	$7.00x10^{-2}$
	N-docosahexaenoyl GABA	0.653	$4.52x10^{-2}$	$8.41x10^{-2}$
	Trans-2, 3, 4-Trimethoxycinnamate	0.604	$2.79x10^{-2}$	$5.80x10^{-2}$
	MG(0:0/18:0/0:0)	0.560	$5.31x10^{-2}$	$9.51x10^{-2}$
	Hexazinone	0.363	$7.24x10^{-2}$	$1.20x10^{-1}$
	Nalorphine	-0.479	$9.30x10^{-2}$	$1.47x10^{-1}$
	Galalpha1-4Galbeta-Cer(d18:1/16:0)	-0.565	$6.72x10^{-2}$	$1.13x10^{-1}$
	(3Z)-Phytochromobilin Esi+8.159999	-0.799	$2.30x10^{-2}$	$4.97x10^{-2}$
	<none> Esi+0.8249996	[illegible]	[illegible]	[illegible]

	C11 H19 N O2	-0.436	8.50x10^{-2}	1.37x10^{-1}
	C31 H57 O9 S	-0.531	6.51x10^{-2}	1.10x10^{-1}
	C28 H41 N8 O3 S	-0.648	6.31x10^{-2}	1.08x10^{-1}
	C17 H29 N O4	-0.732	2.59x10^{-2}	5.47x10^{-2}
	C21 H35 N O4	-1.035	1.05x10^{-4}	1.60x10^{-3}
C18-	2-Thiopheneacrylic acid	0.560	3.17x10^{-3}	1.29x10^{-1}
	PS(13:0/12:0)	-0.870	3.36x10^{-3}	1.29x10^{-1}
	PS(13:0/12:0) Esi-12.402006	-0.960	1.48x10^{-3}	1.29x10^{-1}
	10-hydroxy-2E,8E-Decadiene-4,6-diynoic acid	-1.510	3.69x10^{-4}	6.54x10^{-2}
	<none> Esi-4.960006	0.946	6.87x10^{-4}	9.66x10^{-2}
	C22 H19 N8 O	0.390	3.15x10^{-3}	1.29x10^{-1}
	C41 H69 N3 O12	-0.806	4.05x10^{-3}	1.42x10^{-1}
	C26 H37 N6 O7 Esi-10.69201	-0.824	4.42x10^{-3}	1.43x10^{-1}
	C27 H45 O7	-0.852	3.00x10^{-3}	1.29x10^{-1}
	C22 H49 N17 O4 S	-0.871	3.21x10^{-3}	1.29x10^{-1}
	C28 H55 N19 O2 S	-0.874	4.93x10^{-3}	1.48x10^{-1}
	C32 H63 N6 O9 S	-0.887	2.96x10^{-3}	1.29x10^{-1}
	C33 H55 N3 O9	-0.949	1.79x10^{-3}	1.29x10^{-1}
	C37 H55 O13	-1.168	3.88x10^{-4}	6.54x10^{-2}
	C31 H19 N4 O30 S	-1.183	2.56x10^{-3}	1.29x10^{-1}
	C10 H12 O6 S	-1.772	1.59x10^{-5}	6.69x10^{-3}
	C19 H18 O10 S	-1.988	5.39x10^{-7}	4.55x10^{-4}

FEP_{FU}/HC_{FU} ($\neq FEP_{B}/HC_{B}$ or =- FEP_{B}/HC_{B})

Mode	Metabolomic signatures	Log2 Fold Change (F_{FU}/ H_{FU})	Ave exp	*p*-value
HILIC+	-	-	-	-
HILIC-	AG-041R	0.469	1.15x10^{-3}	5.33x10^{-2}
	Urocanic acid	-0.629	2.08x10^{-3}	7.42x10^{-2}
	C33 H34 N20 O3	-0.366	4.73x10^{-3}	1.41x10^{-1}
	C25 H12 N O7	-1.292	2.27x10^{-3}	7.80x10^{-2}
C18+	Bis (2-hydroxypropyl) amine Esi+0.6719987	0.658	5.22x10^{-2}	8.09x10^{-2}
	Poloxalene	0.362	3.33x10^{-3}	7.65x10^{-3}
	Isobutyrylglycine methyl ester	-0.392	2.40x10^{-2}	4.19x10^{-2}
	Mesuagin	-0.400	3.30x10^{-3}	7.61x10^{-3}
	PC(20:2(11Z,14Z)/0:0)	-0.457	6.89x10^{-3}	1.42x10^{-2}
	Lys Met Lys	-0.484	3.71x10^{-2}	6.12x10^{-2}
	1,3-Dimethyl-6,8-isoquinolinediol	-0.560	1.00x10^{-1}	1.42x10^{-1}
	Theophylline	-0.580	3.84x10^{-3}	8.53x10^{-3}
	Hypoxanthine	-0.660	8.81x10^{-2}	1.27x10^{-1}
	Kurilensoside G	-0.693	1.12x10^{-2}	2.17x10^{-2}
	Myxalamid A	-0.721	5.15x10^{-2}	8.03x10^{-2}
	C16 H30 N7 O2	1.795	4.27x10^{-5}	2.22x10^{-4}
	C20 H49 N17 O4	1.744	9.77x10^{-5}	4.30x10^{-4}
	C20 H41 N8 O4	1.576	1.21x10^{-4}	5.11x10^{-4}
	C17 H32 N7 O3	1.415	6.38x10^{-4}	1.95x10^{-3}
	C13 H24 N7 O2 Esi+5.043013	1.156	2.40x10^{-3}	5.89x10^{-3}
	C13 H24 N7 O2	1.121	3.70x10^{-3}	8.27x10^{-3}
	C12 H22 N7 O	1.036	7.86x10^{-2}	1.16x10^{-1}
	C21 H26 N24 O3	0.511	1.03x10^{-1}	1.45x10^{-1}

	<none> Esi+4.914001	0.495	2.90x10^{-2}	4.93x10^{-2}
	C23 H37 N8	0.491	6.45x10^{-2}	9.73x10^{-2}
	C16 H31 N O4	-0.371	4.44x10^{-3}	9.77x10^{-3}
	C7 H4 O	-0.389	2.60x10^{-2}	4.49x10^{-2}
	C25 H45 N O6	-0.518	8.47x10^{-2}	1.23x10^{-1}
	C13 H32 N12 O4 S	-0.530	4.05x10^{-2}	6.56x10^{-2}
	<none> Esi+6.1630077	-0.575	7.98x10^{-2}	1.17x10^{-1}
	<none> Esi+5.929998	-0.830	3.31x10^{-3}	7.61x10^{-3}
	<none> Esi+6.231005	-0.885	3.36x10^{-3}	7.69x10^{-3}
	C26 H39 N O10	-1.126	2.53x10^{-3}	6.13x10^{-3}
	<none> Esi+5.7470055	-1.215	8.12x10^{-4}	2.38x10^{-3}
	C22 H45 N8 O6 S	-1.245	7.97x10^{-4}	2.35x10^{-3}
	<none> Esi+6.091003	-1.253	2.47x10^{-5}	1.54x10^{-4}
C18-	-	-	-	-

Supplementary Table S14. CSF metabolites differed between follow-up and baseline in healthy controls. Unidentified metabolites are highlighted in gray.

Mode	Metabolomic signatures	Log2 Fold Change (H_{FU}/H_B)	Ave exp	*p*-value	FDR
HILIC+	Deoxymiroestrol	-0.200	9.881	$3.09x10^{-11}$	$3.03X10^{-8}$
	2'-Hydroxy-2,4',6'-trimethoxychalcone	-0.223	9.613	$5.97x10^{-10}$	$2.94X10^{-7}$
	C18 H33 N3 O3	-0.265	7.908	$5.05X10^{-7}$	$6.21X10^{-5}$
HILIC-	Fluconazole	0.291	8.799	$3.77X10^{-7}$	$2.05X10^{-5}$
	3-ethyl-7-isopropyl-1-azulenesulfonate	0.143	10.345	$6.98X10^{-8}$	$5.05X10^{-6}$
	Trinexapac-ethyl	0.112	10.201	$7.36x10^{-11}$	$6.84X10^{-9}$
	N-Formyldemecolcine	-0.061	8.320	$3.94X10^{-2}$	$8.75X10^{-2}$
	C16 H36 N3 O6 S	0.205	8.053	$7.69X10^{-2}$	$1.50X10^{-1}$
	<none> Esi-4.1389923	0.078	10.565	$2.86X10^{-4}$	$4.72X10^{-3}$
	C6 H3 O5 S Esi-1.5169985	0.070	10.405	$1.61X10^{-3}$	$1.12X10^{-2}$
	C27 H26 N23	0.067	10.516	$9.11X10^{-7}$	$4.56X10^{-5}$
	C33 H58 N10	0.056	9.189	$1.34X10^{-4}$	$3.12X10^{-3}$
	C18 H2 N2 O2 S2	-0.056	6.864	$1.30x10^{-12}$	$1.69x10^{-10}$
	C9 H12 N4 O2 S	-0.060	6.942	$2.32x10^{-17}$	$7.57x10^{-15}$
	<none> Esi-0.7699991	-0.095	8.316	$1.24X10^{-3}$	$9.97X10^{-3}$
	187.1203@1.480001	-0.101	8.162	$1.02X10^{-5}$	$4.14X10^{-4}$
	C45 H14 N11 O6	-0.308	7.230	$7.52X10^{-2}$	$1.47X10^{-1}$
	CGP 52608	-0.670	9.233	$1.96X10^{-2}$	$5.05X10^{-2}$
	C40 H6 O5	-0.693	9.403	$1.41X10^{-2}$	$4.09X10^{-2}$
	C21 H2 N3 O4	-1.327	8.732	$1.26X10^{-3}$	$9.99X10^{-3}$
C18+	C29 H59 N8 O4	-0.197	8.682	$6.87X10^{-5}$	$6.83X10^{-2}$
C18-	C5 H3 Cl O7	0.101	8.948	$3.20X10^{-1}$	$8.40X10^{-1}$

Supplementary Table S15. Serum metabolites differed between follow-up and baseline in healthy controls. Unidentified metabolites are highlighted in gray.

Mode	Metabolomic signatures	Log2 Fold Change (H_{FU}/H_{B})	Ave exp	*p*-value	FDR
HILIC+	-	-	-	-	-
HILIC-	Methoxsalen	0.177	9.355	$7.80X10^{-5}$	$6.57X10^{-3}$
	Furmecyclox	0.157	10.020	$4.65x10^{-13}$	$4.31x10^{-10}$
	7Z,11Z,14Z-eicosatrienoic acid	-0.108	9.393	$4.41X10^{-5}$	$4.83X10^{-3}$
	<none> Esi-7.5939946	-0.120	9.361	$3.55X10^{-6}$	$5.47X10^{-4}$
	C25 H12 N O7	-0.296	7.879	$1.47X10^{-4}$	$9.73X10^{-3}$
C18+	Kurilensoside G	0.554	8.620	$1.34X10^{-3}$	$3.89X10^{-3}$
	Pseudoargiopinin III	0.471	8.094	$4.33X10^{-3}$	$9.57X10^{-3}$
	Asp Lys Lys	0.450	8.117	$7.18X10^{-3}$	$1.44X10^{-2}$
	Tetracaine	0.447	8.149	$5.29X10^{-3}$	$1.12X10^{-2}$
	N-acetyl-S-farnesyl-L-Cysteine	0.445	8.151	$4.65X10^{-3}$	$1.01X10^{-2}$
	Thr Lys Lys Esi+5.067995	0.443	8.162	$4.62X10^{-3}$	$1.01X10^{-2}$
	Iodoform	0.428	8.139	$5.72X10^{-3}$	$1.20X10^{-2}$
	1,3-Dimethyl-6,8-isoquinolinediol	0.425	8.163	$4.34X10^{-2}$	$6.41X10^{-2}$
	Serratanidine	0.405	8.097	$1.27X10^{-2}$	$2.24X10^{-2}$
	Arg Gln Ile	0.372	8.229	$1.34X10^{-2}$	$2.33X10^{-2}$
	C23 H32 N11	0.630	8.016	$9.94X10^{-4}$	$3.13X10^{-3}$
	<none> Esi+6.091003	0.584	8.736	$3.95X10^{-3}$	$8.90X10^{-3}$
	C9 H28 N11 O4	0.525	8.095	$1.48X10^{-3}$	$4.20X10^{-3}$
	C34 H45 N13 S	0.483	8.143	$2.17X10^{-3}$	$5.57X10^{-3}$
	C18 H35 N20 O	0.477	8.067	$3.34X10^{-3}$	$7.84X10^{-3}$
	C13 H26 O6	0.474	8.123	$2.28X10^{-3}$	$5.77X10^{-3}$
	C36 H59 N2 O10 S	0.467	8.078	$4.13X10^{-3}$	$9.22X10^{-3}$
	C35 H73 N O17	0.462	8.184	$1.91X10^{-3}$	$5.07X10^{-3}$
	C23 H35 N10	0.446	8.144	$2.97X10^{-3}$	$7.17X10^{-3}$
	<none> Esi+6.098001	0.446	8.129	$6.78X10^{-3}$	$1.38X10^{-2}$
	C18 H37 N8 O4 S	0.442	8.159	$6.11X10^{-3}$	$1.27X10^{-2}$
	C16 H33 N8 O3 Esi+6.3790035	0.439	8.048	$7.35X10^{-3}$	$1.47X10^{-2}$
	C36 H71 N8 O13	0.438	8.142	$2.92X10^{-3}$	$7.07X10^{-3}$
	C27 H45 N2 O8	0.437	8.092	$8.75X10^{-3}$	$1.68X10^{-2}$
	<none> Esi+6.248999	0.436	8.150	$5.23X10^{-3}$	$1.11X10^{-2}$
	C13 H36 N11 O6	0.435	8.193	$3.07X10^{-3}$	$7.33X10^{-3}$
	C26 H41 N2 O S	0.431	8.168	$7.25X10^{-3}$	$1.45X10^{-2}$
	C19 H38 N3 O7	0.425	8.170	$4.62X10^{-3}$	$1.01X10^{-2}$
	<none> Esi+6.096992	0.419	8.222	$5.40X10^{-3}$	$1.14X10^{-2}$
	<none> Esi+6.383996	0.419	8.101	$6.85X10^{-3}$	$1.39X10^{-2}$
	<none> Esi+6.1630077	0.418	8.388	$1.14X10^{-2}$	$2.06X10^{-2}$
	C29 H55 N15 O5	0.416	8.082	$8.87X10^{-3}$	$1.70X10^{-2}$
	C16 H31 N20	0.414	8.165	$8.45X10^{-3}$	$1.64X10^{-2}$
	C17 H35 N15 S	0.412	8.171	$9.69X10^{-3}$	$1.82X10^{-2}$
	C15 H30 O7	0.402	8.201	$3.87X10^{-3}$	$8.75X10^{-3}$
	C17 H34 N3 O6	0.399	8.151	$5.38X10^{-3}$	$1.14X10^{-2}$
	<none> Esi+6.174994	0.395	8.122	$1.10X10^{-2}$	$2.01X10^{-2}$
	C24 H49 N8 O7	0.390	8.158	$9.88X10^{-3}$	$1.85X10^{-2}$
	C36 H71 N5 O13 S	0.389	8.194	$9.81X10^{-3}$	$1.84X10^{-2}$
	C20 H41 N8 O5	0.388	8.185	$1.07X10^{-2}$	$1.97X10^{-2}$

	C28 H57 N8 O9	0.380	8.189	$9.75X10^{-3}$	$1.83X10^{-2}$
	C19 H31 N5 O4	0.374	8.186	$1.09X10^{-2}$	$2.00X10^{-2}$
	C11 H32 N11 O5	0.363	8.213	$7.89X10^{-3}$	$1.55X10^{-2}$
	C23 H33 N2 O5	0.354	8.203	$1.78X10^{-2}$	$2.95X10^{-2}$
	C24 H49 N8 O6	-0.369	7.551	$7.54X10^{-2}$	$1.03X10^{-1}$
	C20 H41 N8 O4	-0.427	7.692	$1.09X10^{-2}$	$2.00X10^{-2}$
	C19 H39 N8 O4	-0.448	7.605	$3.64X10^{-2}$	$5.52X10^{-2}$
	C20 H49 N17 O4	-0.477	7.569	$1.25X10^{-2}$	$2.21X10^{-2}$
	C21 H39 N15	-0.482	7.672	$5.80X10^{-3}$	$1.21X10^{-2}$
C18-	C19 H18 O10 S	-0.273	8.920	$1.28X10^{-5}$	$7.88X10^{-3}$

Supplementary Table S16. Correlations between serum serotonin (5-HT) levels at baseline measured in untargeted metabolomics and various psychiatric and cognitive scale scores.

	**Spearman's *ρ***	***p*-value**
PANSS		
Positive	-0.160	0.445
Negative	-0.052	0.805
General	0.083	0.692
Total	-0.040	0.851
GAF		
Symptoms	0.058	0.784
Functioning	0.012	0.953
CGI	-0.119	0.571
TMT	0.116	0.441
BACS_SC	-0.289	0.051
HVLT_R	-0.154	0.308
WMS_IIISS	-0.002	0.991
LNS	-0.078	0.607
NAB	-0.115	0.446
BVMT_R	-0.169	0.262
Fluency	-0.054	0.723
MSCEIT_ME	0.205	0.176
CPT-IP	-0.156	0.307

Abbreviations: 5-HT,serotonin; PANSS, Positive and Negative Syndrome Scale Score; GAF, Global Assessment of Function; CGI, Clinical Global Impression; TMT, Trail Making Test; BACS-SC, Brief Assessment of Cognition in Schizophrenia-Symbol Coding Subtest; CPT-IP, Continuous Performance Test-Identical Pairs version; LNS, Letter Number Span test; WMS-III SS, Wechsler Memory Scale-3rd ed. Spatial Span subtest; HVLT-R, Hopkins Verbal Learning Test-Revised; NAB, Neuropsychological Assessment Bat.; BVMT-R, Brief Visuospatial Memory Test-Revised; MSCEIT-ME, Mayer-Salovey-Caruso Emotional Intelligence Test-Managing Emotions branch

Supplementary Table S17. Correlations between serum serotonin (5-HT) levels at follow-up measured in untargeted metabolomics and various psychiatric and cognitive scale scores.

	**Spearman's *ρ***	***p*-value**
PANSS		
Positive	-0.126	0.547
Negative	0.239	0.249
General	0.135	0.521
Total	0.160	0.446
GAF		
Symptoms	0.165	0.431
Functioning	0.189	0.366
CGI	-0.169	0.420
TMT	0.070	0.646
BACS_SC	-0.267	0.073
HVLT_R	-0.100	0.510
WMS_IIISS	-0.167	0.268
LNS	-0.150	0.319
NAB	-0.107	0.481
BVMT_R	-0.099	0.513
Fluency	-0.094	0.533
MSCEIT_ME	0.196	0.196
CPT-IP	-0.138	0.367

Abbreviations: 5-HT,serotonin; PANSS, Positive and Negative Syndrome Scale Score; GAF, Global Assessment of Function; CGI, Clinical Global Impression; TMT, Trail Making Test; BACS-SC, Brief Assessment of Cognition in Schizophrenia-Symbol Coding Subtest; CPT-IP, Continuous Performance Test-Identical Pairs version; LNS, Letter Number Span test; WMS-III SS, Wechsler Memory Scale-3rd ed. Spatial Span subtest; HVLT-R, Hopkins Verbal Learning Test-Revised; NAB, Neuropsychological Assessment Bat.; BVMT-R, Brief Visuospatial Memory Test-Revised; MSCEIT-ME, Mayer-Salovey-Caruso Emotional Intelligence Test-Managing Emotions branch

Supplementary Table S18. Correlations between CSF serotonin (5-HT) levels at baseline measured in untargeted metabolomics and various psychiatric and cognitive scale scores.

	**Spearman's *ρ***	***p*-value**
PANSS		
Positive	-0.126	0.549
Negative	-0.035	0.866
General	0.125	0.552
Total	0.001	0.995
GAF		
Symptoms	0.042	0.842
Functioning	0.047	0.825
CGI	-0.157	0.455
TMT	0.170	0.260
BACS_SC	-0.303	0.041
HVLT_R	-0.155	0.305
WMS_IIISS	-0.041	0.785
LNS	-0.036	0.814
NAB	-0.141	0.349
BVMT_R	-0.215	0.151
Fluency	-0.053	0.726
MSCEIT_ME	0.237	0.118
CPT-IP	-0.160	0.293

Abbreviations: 5-HT,serotonin; PANSS, Positive and Negative Syndrome Scale Score; GAF, Global Assessment of Function; CGI, Clinical Global Impression; TMT, Trail Making Test; BACS-SC, Brief Assessment of Cognition in Schizophrenia-Symbol Coding Subtest; CPT-IP, Continuous Performance Test-Identical Pairs version; LNS, Letter Number Span test; WMS-III SS, Wechsler Memory Scale-3rd ed. Spatial Span subtest; HVLT-R, Hopkins Verbal Learning Test-Revised; NAB, Neuropsychological Assessment Bat.; BVMT-R, Brief Visuospatial Memory Test-Revised; MSCEIT-ME, Mayer-Salovey-Caruso Emotional Intelligence Test-Managing Emotions branch

Supplementary Table S19. Correlations between CSF serotonin (5-HT) levels at follow-up measured in untargeted metabolomics and various psychiatric and cognitive scale scores.

	**Spearman's *ρ***	***p*-value**
PANSS		
Positive	-0.151	0.471
Negative	0.110	0.599
General	0.168	0.423
Total	0.089	0.672
GAF		
Symptoms	-0.035	0.870
Functioning	-0.143	0.494
CGI	0.006	0.976
TMT	-0.024	0.876
BACS_SC	-0.184	0.220
HVLT_R	0.023	0.878
WMS_IIISS	-0.142	0.346
LNS	-0.095	0.529
NAB	-0.109	0.472
BVMT_R	-0.062	0.684
Fluency	-0.046	0.759
MSCEIT_ME	0.215	0.156
CPT-IP	-0.056	0.713

Abbreviations: 5-HT,serotonin; PANSS, Positive and Negative Syndrome Scale Score; GAF, Global Assessment of Function; CGI, Clinical Global Impression; TMT, Trail Making Test; BACS-SC, Brief Assessment of Cognition in Schizophrenia-Symbol Coding Subtest; CPT-IP, Continuous Performance Test-Identical Pairs version; LNS, Letter Number Span test; WMS-III SS, Wechsler Memory Scale-3rd ed. Spatial Span subtest; HVLT-R, Hopkins Verbal Learning Test-Revised; NAB, Neuropsychological Assessment Bat.; BVMT-R, Brief Visuospatial Memory Test-Revised; MSCEIT-ME, Mayer-Salovey-Caruso Emotional Intelligence Test-Managing Emotions branch

Supplementary Table S20. Correlations between the change in serum serotonin (5-HT) levels after 18-month treatment measured in untargeted metabolomics and various psychiatric and cognitive scale scores.

	**Spearman's *ρ***	***p*-value**
PANSS		
Positive	-0.374	0.066
Negative	-0.336	0.101
General	-0.157	0.454
Total	-0.299	0.147
GAF		
Symptoms	-0.318	0.140
Functioning	-0.388	0.067
CGI	0.080	0.712
TMT	0.184	0.379
BACS_SC	-0.011	0.958
HVLT_R	-0.101	0.632
WMS_IIISS	0.248	0.232
LNS	-0.160	0.445
NAB	0.042	0.841
BVMT_R	0.095	0.652
Fluency	0.006	0.979
MSCEIT_ME	-0.311	0.139
CPT-IP	-0.022	0.918

Abbreviations: 5-HT,serotonin; PANSS, Positive and Negative Syndrome Scale Score; GAF, Global Assessment of Function; CGI, Clinical Global Impression; TMT, Trail Making Test; BACS-SC, Brief Assessment of Cognition in Schizophrenia-Symbol Coding Subtest; CPT-IP, Continuous Performance Test-Identical Pairs version; LNS, Letter Number Span test; WMS-III SS, Wechsler Memory Scale-3rd ed. Spatial Span subtest; HVLT-R, Hopkins Verbal Learning Test-Revised; NAB, Neuropsychological Assessment Bat.; BVMT-R, Brief Visuospatial Memory Test-Revised; MSCEIT-ME, Mayer-Salovey-Caruso Emotional Intelligence Test-Managing Emotions branch

Supplementary Table S21. Correlations between the change in CSF serotonin (5-HT) levels after 18-month treatment measured in untargeted metabolomics and various psychiatric and cognitive scale scores.

	**Spearman's *ρ***	***p*-value**
PANSS		
Positive	-0.246	0.235
Negative	-0.390	0.054
General	-0.425	0.034
Total	-0.448	0.025
GAF		
Symptoms	0.072	0.744
Functioning	0.017	0.938
CGI	-0.050	0.817
TMT	0.155	0.459
BACS_SC	-0.149	0.477
HVLT_R	-0.196	0.347
WMS_IIISS	0.177	0.398
LNS	0.122	0.561
NAB	-0.048	0.820
BVMT_R	-0.003	0.990
Fluency	-0.242	0.244
MSCEIT_ME	-0.472	0.020
CPT-IP	-0.192	0.369

Abbreviations: 5-HT,serotonin; PANSS, Positive and Negative Syndrome Scale Score; GAF, Global Assessment of Function; CGI, Clinical Global Impression; TMT, Trail Making Test; BACS-SC, Brief Assessment of Cognition in Schizophrenia-Symbol Coding Subtest; CPT-IP, Continuous Performance Test-Identical Pairs version; LNS, Letter Number Span test; WMS-III SS, Wechsler Memory Scale-3rd ed. Spatial Span subtest; HVLT-R, Hopkins Verbal Learning Test-Revised; NAB, Neuropsychological Assessment Bat.; BVMT-R, Brief Visuospatial Memory Test-Revised; MSCEIT-ME, Mayer-Salovey-Caruso Emotional Intelligence Test-Managing Emotions branch

Supplementary Table S22. Effects of age on CSF metabolites differed between FEP patients and healthy controls at baseline. Unidentified metabolites are highlighted in gray. Note that statistical significance was defined as FDR < 0.15 with corresponding modes' fold change thresholds.

Mode	Metabolomic signatures	Log2 Fold Change	Ave exp	*p*-value	FDR
HILIC+	Acanthicifoline	0.083	6.938	$1.49X10^{-5}$	$2.09X10^{-3}$
	Guvacoline	-0.012	7.855	$6.09X10^{-1}$	$8.72X10^{-1}$
	Serotonin	0.081	7.694	$2.34X10^{-12}$	$2.30X10^{-9}$
	Pyriculol	-0.036	8.038	$1.31X10^{-1}$	$4.65X10^{-1}$
	Athamantin	-0.002	9.639	$6.25X10^{-1}$	$8.80X10^{-1}$
	C6 H18 N2 O2	0.027	8.923	$3.33X10^{-2}$	$2.48X10^{-1}$
HILIC-	Citric acid	-0.029	9.317	$1.85X10^{-1}$	$4.75X10^{-1}$
	Acetylenedicarboxylate	0.000	11.257	$7.26X10^{-1}$	$9.10X10^{-1}$
	Pyriculol	-0.002	10.757	$2.11X10^{-2}$	$1.65X10^{-1}$
	Rhamnetin 3-(3''''-p-coumaryl-rhamnosyl)(1^{-3})-rhamnosyl-(1-6)-galactoside	-0.007	9.266	$7.79X10^{-1}$	$9.16X10^{-1}$
C18+	Serotonin	0.073	7.101	$2.80X10^{-4}$	$3.10X10^{-2}$
C18-	-	-	-	-	-

Supplementary Table S23. Effects of age on serum metabolites differed between FEP patients and healthy controls at baseline. Unidentified metabolites are highlighted in gray. Note that statistical significance was defined as FDR < 0.15 with corresponding modes' fold change thresholds.

Mode	Metabolomic signatures	Log2 Fold Change	Ave exp	*p*-value	FDR
HILIC+	-	-	-	-	-
HILIC-	N-depyridomethyl-Indinavir	-0.002	9.751	5.47X10^{-1}	6.85X10^{-1}
	Norathyriol	-0.007	7.444	7.95X10^{-1}	8.63X10^{-1}
	C13 H13 N8 O3 S2	0.005	7.760	8.48X10^{-1}	8.97X10^{-1}
	C5 H5 N O4 S	0.002	10.153	1.72X10^{-1}	2.93X10^{-1}
	C11 H3 N O12 S2	0.007	9.841	5.45X10^{-3}	2.56X10^{-2}
	C31 H44 N2 O5	-0.006	9.761	9.35X10^{-2}	1.90X10^{-1}
	C19 H3 N3 O7	-0.063	9.039	5.75X10^{-3}	2.63X10^{-2}
	C33 H56 N3 O7	0.004	9.331	4.64X10^{-1}	6.18X10^{-1}
	C34 H25 N26	-0.013	7.615	6.29X10^{-1}	7.40X10^{-1}
C18+	Myxalamid A	-0.008	7.911	7.01X10^{-1}	8.20X10^{-1}
	N-oleoyl methionine	0.023	7.811	2.98X10^{-1}	5.75X10^{-1}
	Val Val	-0.022	8.498	2.42X10^{-1}	5.33X10^{-1}
	PE(20:5(5Z,8Z,11Z,14Z,17Z)/0:0)	0.019	9.016	8.00X10^{-2}	3.68X10^{-1}
	17-Methyl^{-1}8-norandrosta-4,13(17)-dien-3-one	-0.020	8.013	3.40X10^{-1}	6.12X10^{-1}
	N-docosahexaenoyl GABA	0.017	8.899	1.39X10^{-1}	4.29X10^{-1}
	Trans-2, 3, 4-Trimethoxycinnamate	0.003	9.218	4.20X10^{-1}	6.68X10^{-1}
	S-Farnesyl Thioacetic Acid	0.005	8.532	8.04X10^{-1}	8.87X10^{-1}
	MG(0:0/18:0/0:0)	0.003	9.290	3.20X10^{-1}	5.92X10^{-1}
	Hexazinone	0.004	9.574	6.53X10^{-1}	8.05X10^{-1}
	estrone 3-sulfate	0.004	9.366	1.68X10^{-1}	4.70X10^{-1}
	Nalorphine	-0.003	8.923	9.30X10^{-1}	9.63X10^{-1}
	Galalpha1-4Galbeta-Cer(d18:1/16:0)	-0.046	9.252	8.96X10^{-2}	3.72X10^{-1}
	Theobromine Esi+3.7969975	0.065	8.394	1.44X10^{-8}	9.20X10^{-6}
	(3Z)-Phytochromobilin Esi+8.159999	0.033	8.714	1.04X10^{-4}	7.37X10^{-3}
	Tetracaine	-0.011	7.046	6.09X10^{-1}	7.87X10^{-1}
	Serratanidine	-0.012	7.046	5.11X10^{-1}	7.37X10^{-1}
	N-acetyl-S-farnesyl-L-Cysteine	-0.011	7.069	5.66X10^{-1}	7.65X10^{-1}
	Iodoform	-0.013	7.075	4.95X10^{-1}	7.26X10^{-1}
	Pseudopelletierine	-0.011	7.210	5.63X10^{-1}	7.63X10^{-1}
	Asp Lys Lys	-0.009	7.016	6.54X10^{-1}	8.05X10^{-1}
	Lysyl-Tyrosyl-Lysine	-0.011	7.136	5.46X10^{-1}	7.55X10^{-1}
	Pseudoargiopinin III	-0.011	6.952	5.73X10^{-1}	7.65X10^{-1}
	Arg Gln Ile	-0.010	7.080	5.96X10^{-1}	7.81X10^{-1}
	Thr Lys Lys Esi+5.067995	-0.012	7.027	5.34X10^{-1}	7.45X10^{-1}
	Cadiamine	-0.012	7.177	5.52X10^{-1}	7.56X10^{-1}
	Gabapentin	-0.008	7.060	6.97X10^{-1}	8.19X10^{-1}
	C25 H28 N9 O2 S	0.018	6.695	2.84X10^{-1}	5.59X10^{-1}
	C24 H49 N8 O6	0.029	8.638	1.07X10^{-3}	3.67X10^{-2}
	C19 H39 N8 O4	0.022	8.783	4.71X10^{-4}	2.40X10^{-2}
	C16 H33 N8 O3	0.024	8.793	2.63X10^{-4}	1.60X10^{-2}
	C21 H39 N15	0.026	8.800	1.29X10^{-3}	4.23X10^{-2}
	<none> Esi+0.8249996	-0.021	7.573	3.18X10^{-1}	5.92X10^{-1}
	<none> Esi+4.9589953	0.020	9.066	1.15X10^{-2}	1.48X10^{-1}

C15 H28 N7 O2	0.025	8.852	9.51X10^{-4}	3.57X10^{-2}
C11 H19 N O2	0.016	9.011	1.67X10^{-1}	4.70X10^{-1}
C31 H57 O9 S	-0.035	8.916	6.84X10^{-2}	3.44X10^{-1}
C28 H41 N8 O3 S	-0.002	7.228	9.29X10^{-1}	9.63X10^{-1}
C17 H29 N O4	0.055	8.490	6.89X10^{-10}	8.78X10^{-7}
C36 H59 N2 O10 S	-0.008	7.036	6.80X10^{-1}	8.09X10^{-1}
C13 H26 O6	-0.008	7.058	6.76X10^{-1}	8.09X10^{-1}
C34 H45 N13 S	-0.011	7.082	5.52X10^{-1}	7.56X10^{-1}
<none> Esi+6.316997	-0.010	7.163	5.69X10^{-1}	7.65X10^{-1}
C35 H73 N O17	-0.012	7.072	5.27X10^{-1}	7.44X10^{-1}
C21 H35 N O4	0.017	8.788	2.93X10^{-2}	2.51X10^{-1}
C9 H28 N11 O4	-0.011	6.976	5.71X10^{-1}	7.65X10^{-1}
<none> Esi+6.248999	-0.010	7.065	6.12X10^{-1}	7.88X10^{-1}
C20 H41 N8 O5	-0.012	7.109	5.19X10^{-1}	7.38X10^{-1}
<none> Esi+6.096992	-0.011	7.123	5.54X10^{-1}	7.57X10^{-1}
<none> Esi+6.098001	-0.012	7.033	5.15X10^{-1}	7.38X10^{-1}
C21 H35 N3 O4	-0.002	7.266	9.20X10^{-1}	9.58X10^{-1}
C33 H53 N5 O6 Esi+5.3609943	-0.011	7.130	5.80X10^{-1}	7.71X10^{-1}
<none> Esi+5.583006	-0.010	7.213	6.04X10^{-1}	7.86X10^{-1}
C23 H49 N O11	-0.010	7.118	6.40X10^{-1}	7.99X10^{-1}
C16 H31 N20	-0.011	7.075	5.69X10^{-1}	7.65X10^{-1}
C28 H57 N8 O9	-0.011	7.101	5.72X10^{-1}	7.65X10^{-1}
C19 H31 N5 O4	-0.011	7.115	5.47X10^{-1}	7.55X10^{-1}
C18 H35 N20 O	-0.012	6.959	5.37X10^{-1}	7.49X10^{-1}
C33 H69 N O16	-0.011	7.101	5.64X10^{-1}	7.63X10^{-1}
C17 H37 N O8	-0.008	7.211	6.58X10^{-1}	8.05X10^{-1}
C21 H35 N3 O4 Esi+5.0669937	-0.005	7.153	8.10X10^{-1}	8.90X10^{-1}
C26 H41 N2 O S	-0.014	7.060	4.63X10^{-1}	7.04X10^{-1}
C23 H33 N2 O5	-0.012	7.116	5.08X10^{-1}	7.34X10^{-1}
C27 H49 N12 O4	-0.012	7.175	5.33X10^{-1}	7.45X10^{-1}
<none> Esi+6.174994	-0.013	7.036	4.98X10^{-1}	7.26X10^{-1}
C36 H71 N8 O13	-0.012	7.046	5.27X10^{-1}	7.44X10^{-1}
C17 H35 N15 S	-0.011	7.041	5.70X10^{-1}	7.65X10^{-1}
C16 H33 N8 O3 Esi+6.3790035	-0.011	6.945	5.67X10^{-1}	7.65X10^{-1}
C23 H35 N10	-0.005	7.060	7.82X10^{-1}	8.74X10^{-1}
C36 H71 N5 O13 S	-0.010	7.101	5.97X10^{-1}	7.81X10^{-1}
<none> Esi+6.383996	-0.009	7.025	6.23X10^{-1}	7.93X10^{-1}
C19 H38 N3 O7	-0.008	7.023	7.02X10^{-1}	8.20X10^{-1}
C11 H32 N11 O5	-0.010	7.101	6.11X10^{-1}	7.88X10^{-1}
C15 H30 O7	-0.008	7.074	6.61X10^{-1}	8.06X10^{-1}
C17 H34 N3 O6	-0.014	7.027	4.75X10^{-1}	7.11X10^{-1}
C13 H36 N11 O6	-0.010	7.071	5.84X10^{-1}	7.73X10^{-1}
C27 H45 N2 O8	-0.009	6.994	6.48X10^{-1}	8.03X10^{-1}
C30 H61 N8 O10 S	-0.011	7.210	5.64X10^{-1}	7.63X10^{-1}
C17 H2 N2 O18 S2	-0.013	7.159	5.32X10^{-1}	7.44X10^{-1}
C18 H37 N8 O4 S	-0.013	6.961	5.39X10^{-1}	7.50X10^{-1}
C33 H61 N12 O7 S	-0.010	7.092	6.16X10^{-1}	7.91X10^{-1}
C22 H45 N8 O6	-0.013	7.111	5.04X10^{-1}	7.31X10^{-1}
C29 H53 N12 O5 S	-0.007	7.097	7.24X10^{-1}	8.35X10^{-1}
C29 H55 N15 O5	-0.014	6.943	4.81X10^{-1}	7.15X10^{-1}
C30 H59 N8 O10	-0.010	7.074	6.20X10^{-1}	7.92X10^{-1}
C27 H57 N O13	-0.014	7.074	4.80X10^{-1}	7.15X10^{-1}
C33 H53 N5 O6	-0.019	7.037	3.76X10^{-1}	6.36X10^{-1}
C28 H55 N5 O9 S	-0.020	7.039	3.41X10^{-1}	6.12X10^{-1}
C23 H32 N11	0.009	6.780	6.06X10^{-1}	7.86X10^{-1}
C19 H41 N O9	-0.010	7.091	6.20X10^{-1}	7.92X10^{-1}

	C23 H N2 O21	-0.015	7.128	$4.70X10^{-1}$	$7.07X10^{-1}$
	C24 H49 N8 O7	-0.013	6.991	$5.18X10^{-1}$	$7.38X10^{-1}$
	C8 Cl3 N3 O S5	-0.008	7.067	$6.95X10^{-1}$	$8.19X10^{-1}$
	C20 H33 N3 O S	-0.011	7.081	$5.80X10^{-1}$	$7.71X10^{-1}$
	C12 H29 N9 O4	-0.012	7.089	$5.50X10^{-1}$	$7.56X10^{-1}$
	C29 H61 N O14	-0.009	7.062	$6.43X10^{-1}$	$8.01X10^{-1}$
	C25 H53 N O12	-0.007	7.016	$7.26X10^{-1}$	$8.36X10^{-1}$
	C21 H45 N O10	-0.015	7.020	$4.52X10^{-1}$	$6.94X10^{-1}$
	C15 H33 N13 O4	-0.020	7.137	$3.04X10^{-1}$	$5.83X10^{-1}$
C18-	2-Thiopheneacrylic acid	0.001	10.133	$5.33X10^{-1}$	$6.62X10^{-1}$
	C27 H45 O7	-0.018	9.650	$5.10X10^{-1}$	$6.41X10^{-1}$
	PS(13:0/12:0)	-0.013	9.685	$7.32X10^{-1}$	$8.30X10^{-1}$
	PS(13:0/12:0) Esi^{-1}2.402006	-0.014	9.619	$7.18X10^{-1}$	$8.24X10^{-1}$
	10-hydroxy-2E,8E-Decadiene-4,6-diynoic acid	-0.009	8.506	$7.31X10^{-1}$	$8.30X10^{-1}$
	<none> Esi-4.960006	0.002	9.732	$5.91X10^{-1}$	$7.08X10^{-1}$
	C22 H19 N8 O	-0.005	10.221	$1.39X10^{-1}$	$2.21X10^{-1}$
	C41 H69 N3 O12	0.009	9.670	$6.90X10^{-1}$	$8.02X10^{-1}$
	C26 H37 N6 O7 Esi^{-1}0.69201	0.029	9.429	$7.76X10^{-2}$	$1.34X10^{-1}$
	C22 H49 N17 O4 S	0.021	9.583	$3.32X10^{-1}$	$4.55X10^{-1}$
	C28 H55 N19 O2 S	0.034	9.473	$3.50X10^{-2}$	$6.81X10^{-2}$
	C32 H63 N6 O9 S	0.027	9.504	$1.09X10^{-1}$	$1.82X10^{-1}$
	C33 H55 N3 O9	-0.017	9.633	$6.78X10^{-1}$	$7.91X10^{-1}$
	C37 H55 O13	0.005	9.288	$9.08X10^{-1}$	$9.42X10^{-1}$
	C31 H19 N4 O30 S	0.012	8.539	$6.19X10^{-1}$	$7.36X10^{-1}$
	C10 H12 O6 S	-0.015	8.340	$5.55X10^{-1}$	$6.80X10^{-1}$
	C19 H18 O10 S	-0.056	7.983	$1.78X10^{-2}$	$3.78X10^{-2}$
