# Supplementary material for: Identification of cerebrospinal fluid and serum metabolomic biomarkers in first episode psychosis patients
Source: Transl Psychiatry. 2022 Jun 3;12:229. doi: 10.1038/s41398-022-02000-1 (PMC9166796; doi:10.1038/s41398-022-02000-1)
Supplement: Supplementary file 4 — Supplemental Figures [file 41398_2022_2000_MOESM4_ESM.pdf]

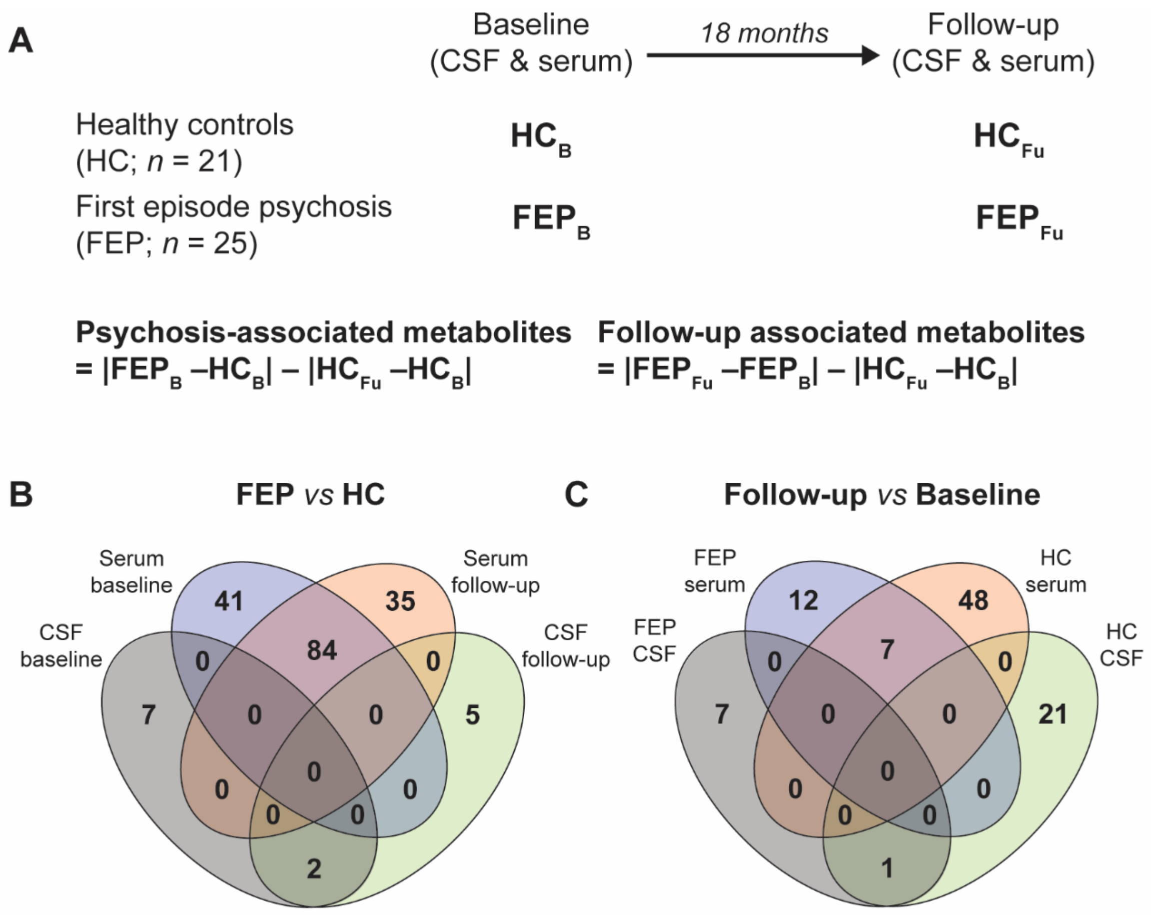

Supplementary Figures S1. Study design and the number of metabolites in cerebrospinal fluid (CSF) and serum that are associated with first-episode psychosis (FEP) and 18-month follow-up. **(A)** CSF and serum samples were collected from 25 FEP patients and 21 healthy controls at baseline (i.e., diagnosis of FEP for patients and prior to follow-up) and 18 months later at a follow-up visit. The CSF or serum metabolomes at baseline for FEP patients and controls were abbreviated as FEP_B and HC_B respectively, while the CSF or serum metabolomes at follow up were abbreviated as FEP_{Fu} and HC_{Fu} respectively. The definitions of psychosis and follow-up associated metabolites are stated respectively. Note that apart from the general comparisons where the psychosis and follow-up associated metabolites are defined, some metabolites were removed to account for the effect of aging and/or additional metabolites were included. **(B)** Venn diagram for the number of metabolites detected in CSF and serum which were significantly different between time points by groups. **(C)** Venn diagram for the number of metabolites detected in CSF and serum which were significantly different between groups by time points. (Abbreviations: CSF, cerebrospinal fluid; FEP, first episode psychosis; FEP_B, metabolites detected at baseline in FEP patients; FEP_{Fu}, metabolites detected at follow up in FEP patients; HC_B, metabolites detected at baseline in healthy controls; HC_{Fu}, metabolites detected at follow up in healthy controls).

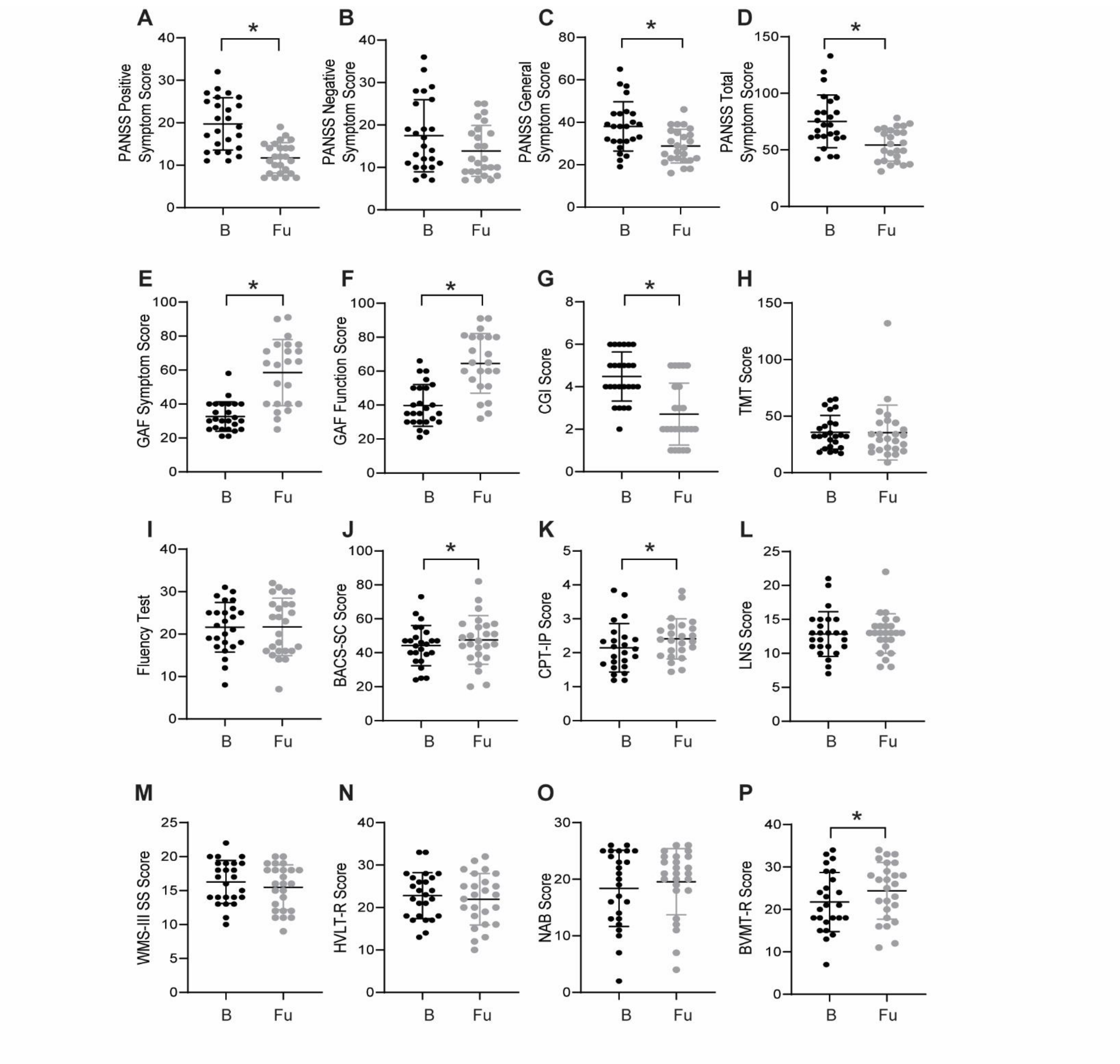

Supplementary Figure S2. Comparisons of behavior test results of patients with FEP between baseline and 1.5-year follow-up. **(A-D)** PANSS scores of patients with FEP at baseline and 1.5-year follow-up. **(E-G)** GAF and CGI scores. **(H-P)** Cognitive characteristics evaluated by TMT, Fluency Test, BACS-SC, CPT-IP, LNS, WMS-III SS, HVLT-R, NAB, and BVMT-R tests. (Abbreviations: PANSS, Positive and Negative Syndrome Scale Score; GAF, Global Assessment of Function; CGI, Clinical Global Impression; TMT, Trail Making Test; BACS-SC, Brief Assessment of Cognition in Schizophrenia-Symbol Coding Subtest; CPT-IP, Continuous Performance Test-Identical Pairs version; LNS, Letter Number Span test; WMS-III SS, Wechsler Memory Scale-3rd ed. Spatial Span subtest; HVLT-R, Hopkins Verbal Learning Test-Revised; NAB, Neuropsychological Assessment Bat.; BVMT-R, Brief Visuospatial Memory Test-Revised).

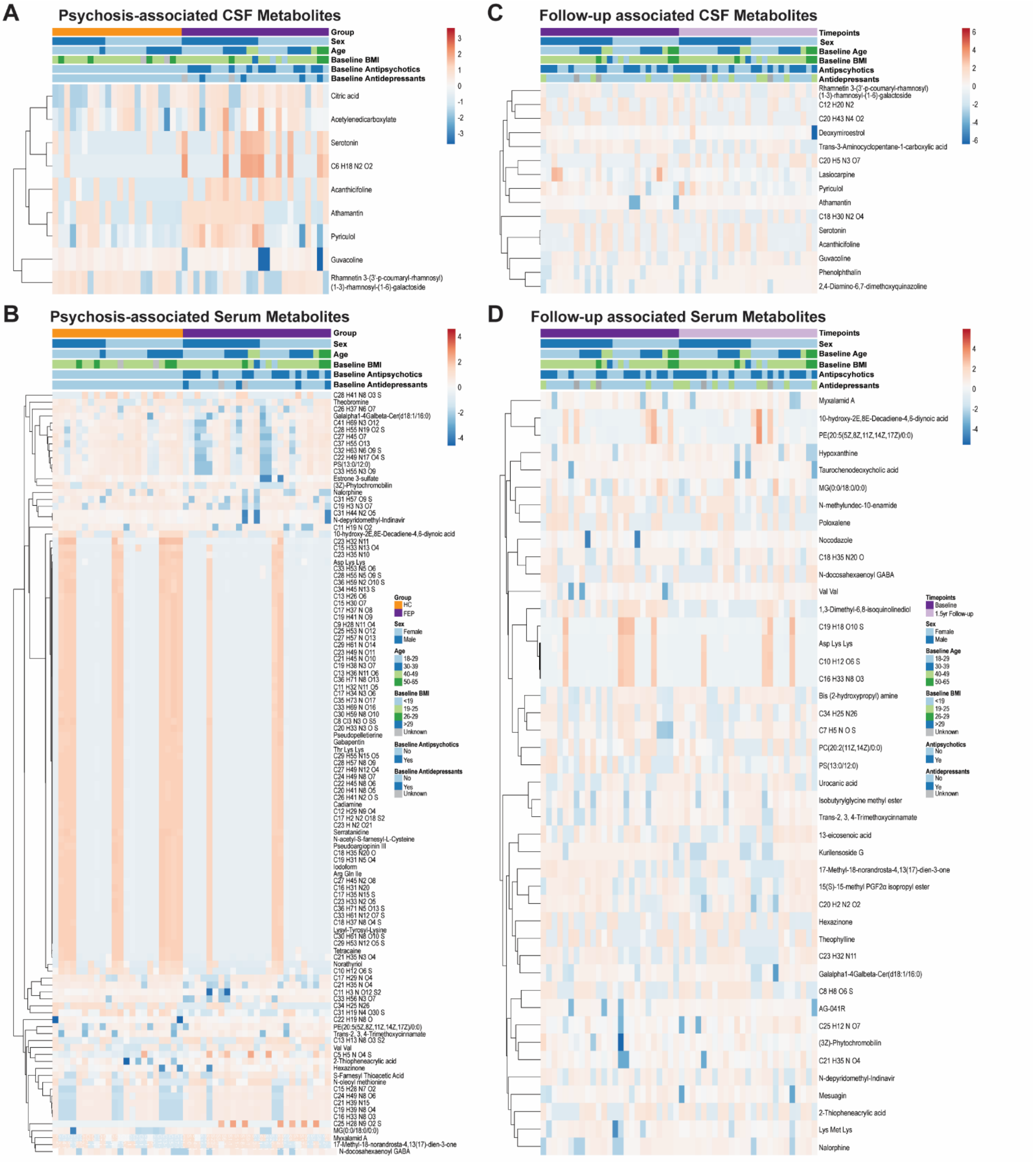

Supplementary Figure S3. Heatmaps for psychosis and follow-up associated metabolite levels overlay with subject demographics and clinical characteristics. **(A)** Psychosis-associated CSF metabolites. (**B**) Psychosis-associated serum metabolites. **(C)** Follow-up associated CSF metabolites. **(D)** Follow-up associated serum metabolites. Full lists of these metabolites are in Supplementary Table S14-17. Heatmaps were generated by ClustVis using normalized Log metabolite levels. Metabolite hierarchical clustering was performed with Pearson's correlation as a measure of dissimilarity.

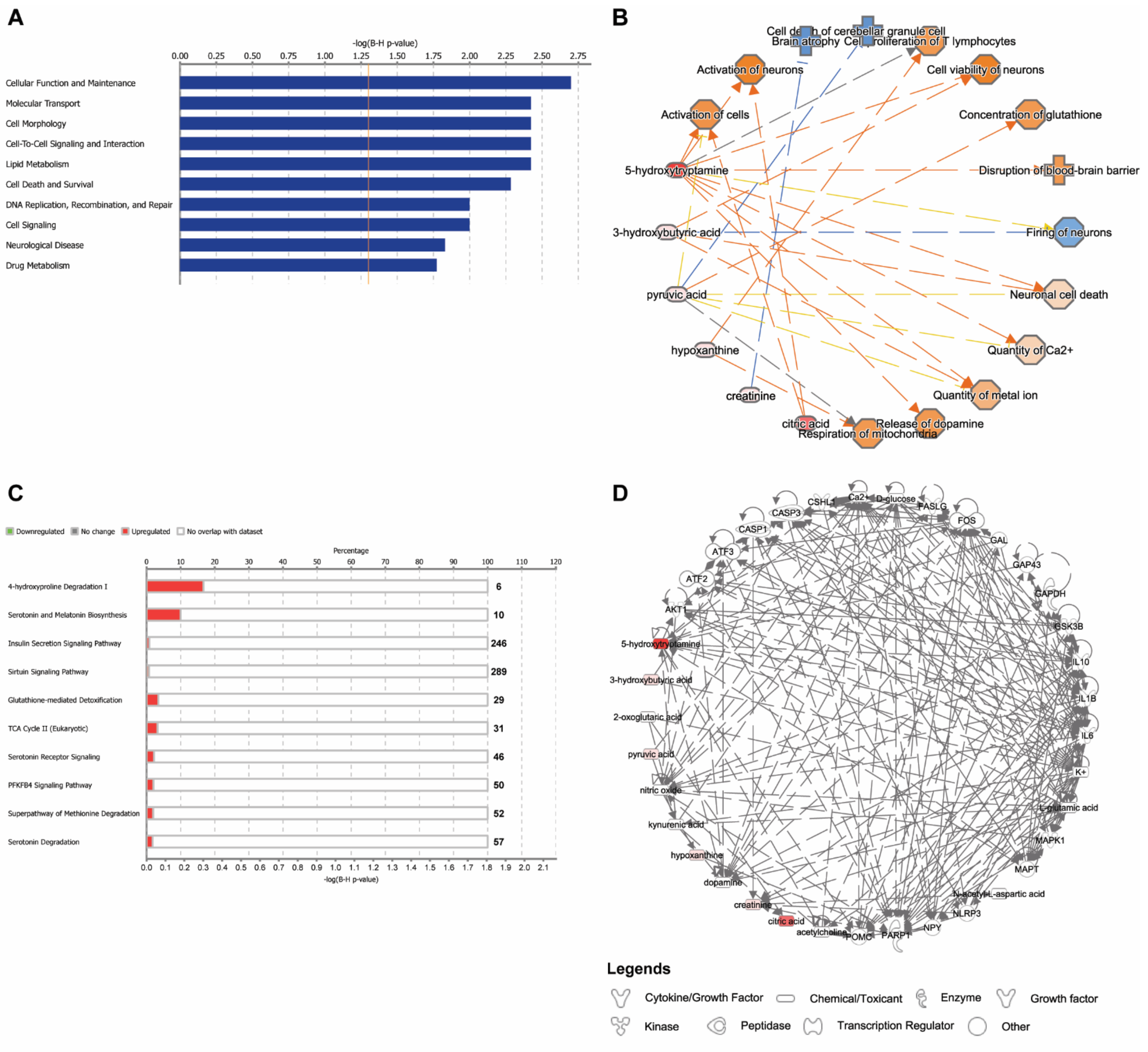

Supplementary Figures S4. IPA results of CSF psychosis-associated metabolites. Using CSF psychosis-associated metabolites, we identified **(A)** top enriched biofunction and disease pathways. **(B)** Top networks involved in disease pathways. **(C)** Top canonical pathways. **(D)** Top networks involved in diseases and functions. In **(B)**, red represents increased measurement, green represents decreased measurement, orange color and orange line represents predicted activation, blue color and blue line represents predicted inhibition, yellow line represents findings inconsistent with the state of downstream molecule, grey represents effect not predicted. In **(D)**, red represents increased measurement, green represents decreased measurement.

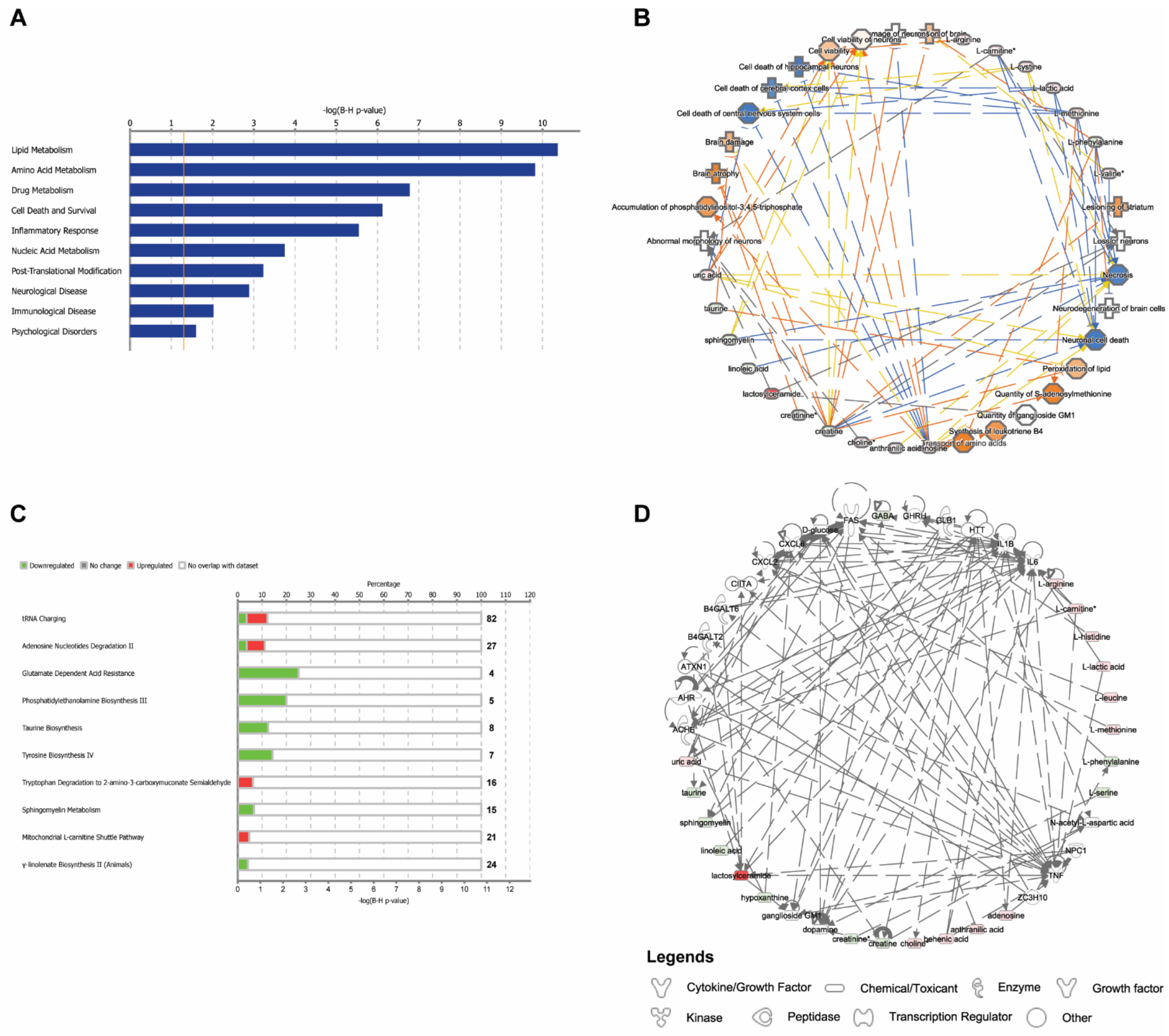

Supplementary Figures S5. IPA results of serum psychosis-associated metabolites. Using serum psychosis-associated metabolites, we identified **(A)** top enriched biofunction and disease pathways. **(B)** Top networks involved in disease pathways. **(C)** Top canonical pathways. **(D)** Top networks involved in diseases and functions. In **(B)**, red represents increased measurement, green represents decreased measurement, orange color and orange line represents predicted activation, blue color and blue line represents predicted inhibition, yellow line represents findings inconsistent with the state of downstream molecule, grey represents effect not predicted. In **(D)**, red represents increased measurement, green represents decreased measurement.

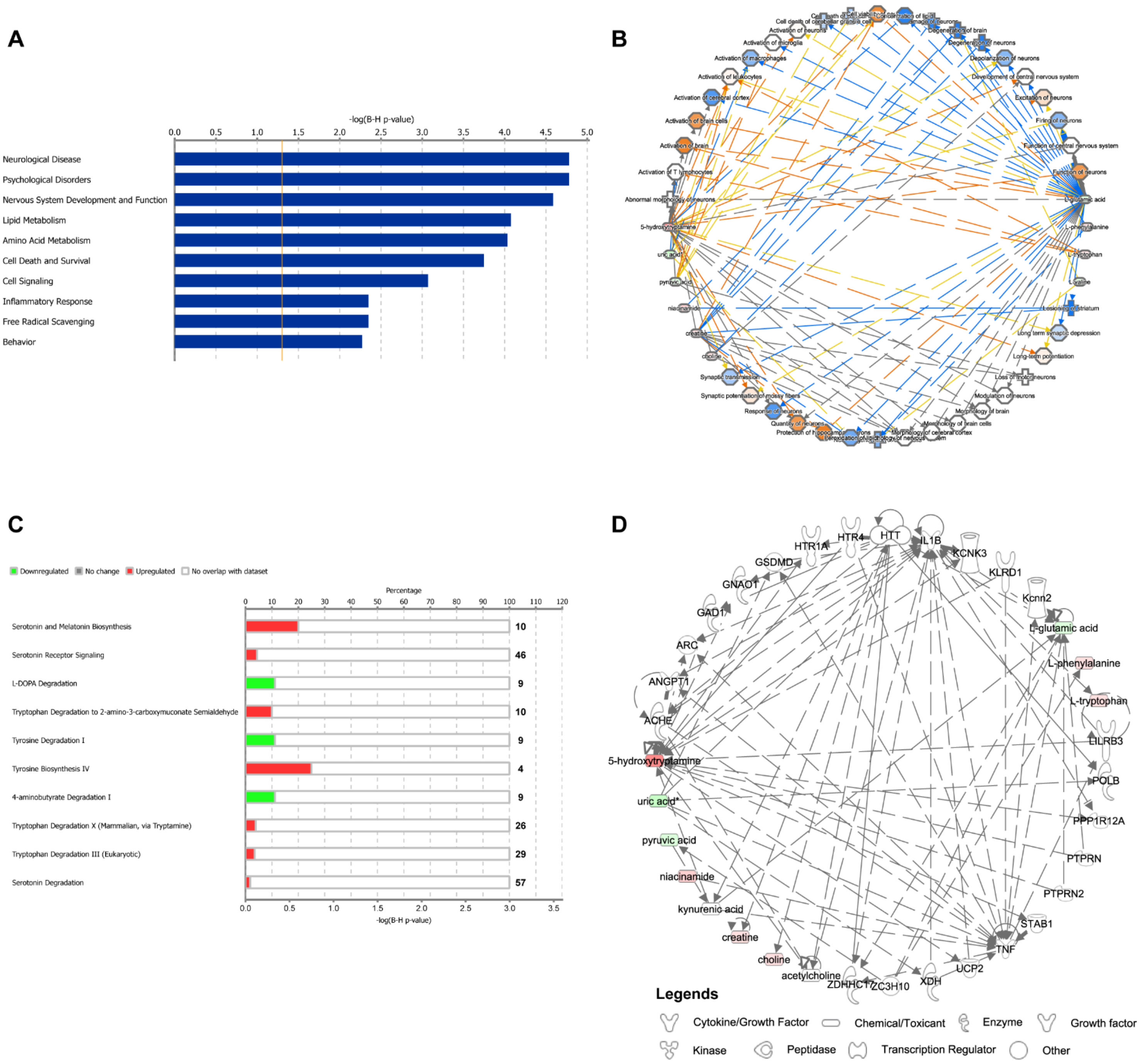

Supplementary Figures S6. IPA results of CSF follow-up associated metabolites. Using CSF follow-up associated metabolites, we identified **(A)** top enriched biofunction and disease pathways. **(B)** Top networks involved in disease pathways. **(C)** Top canonical pathways. **(D)** Top networks involved in diseases and functions. In **(B)**, red represents increased measurement, green represents decreased measurement, orange color and orange line represents predicted activation, blue color and blue line represents predicted inhibition, yellow line represents findings inconsistent with the state of downstream molecule, grey represents effect not predicted. In **(D)**, red represents increased measurement, green represents decreased measurement.

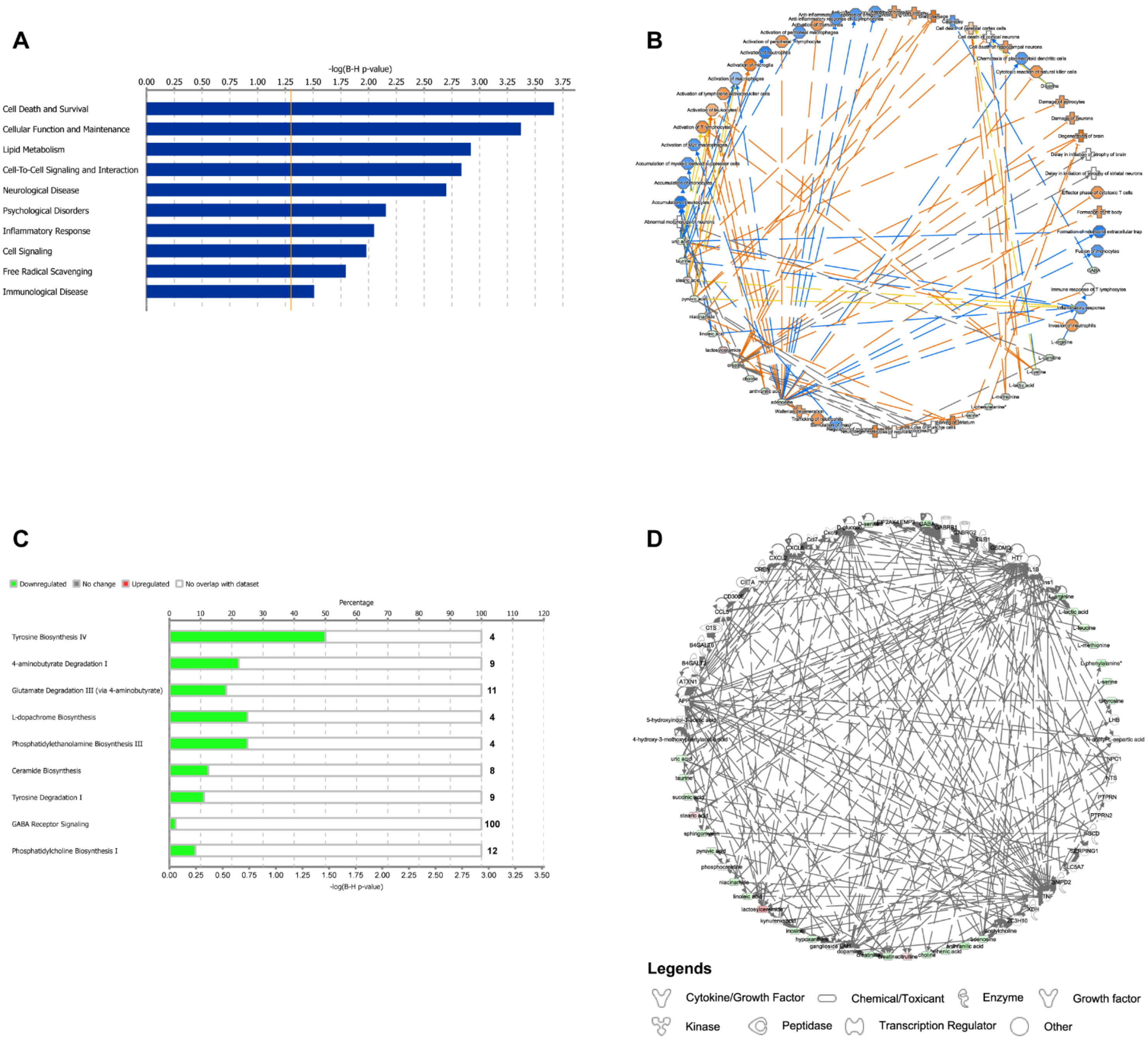

Supplementary Figures S7. IPA results of serum follow-up associated metabolites. Using serum follow-up associated metabolites, we identified **(A)** top enriched biofunction and disease pathways. **(B)** Top networks involved in disease pathways. **(C)** Top canonical pathways. **(D)** Top networks involved in diseases and functions. In **(B)**, red represents increased measurement, green represents decreased measurement, orange color and orange line represents predicted activation, blue color and blue line represents predicted inhibition, yellow line represents findings inconsistent with the state of downstream molecule, grey represents effect not predicted. In **(D)**, red represents increased measurement, green represents decreased measurement.

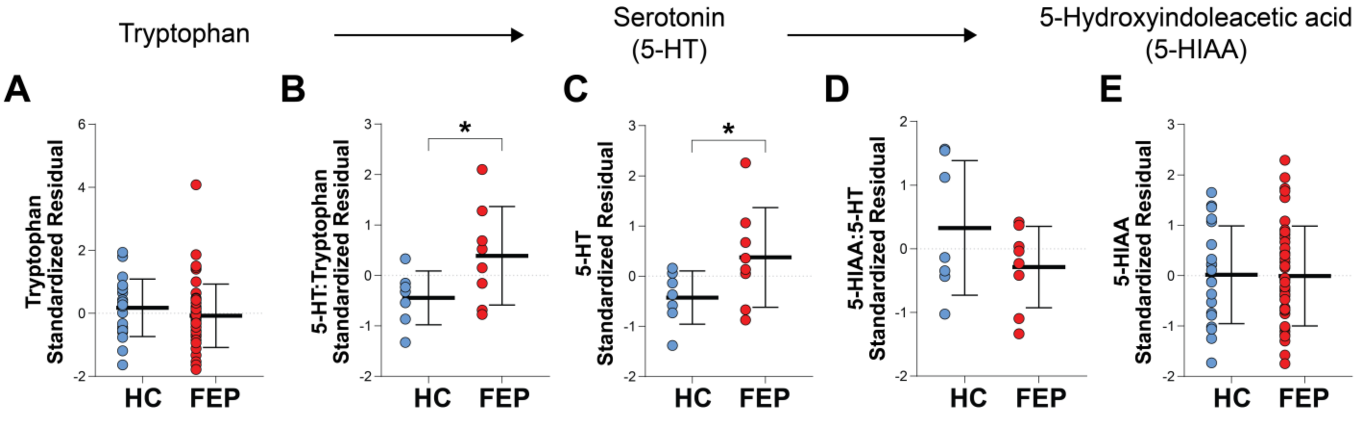

Supplementary Table S8. Standardized residuals (mean ± SD) of serotonin-related metabolites after adjusting for age, antidepressant use, and nicotine use. Asterisks note the differences between healthy controls (HC) and first-episode psychosis (FEP) patients at $p_{unadjusted} < 0.05$ accounting for the aforementioned covariates. (**A**) Tryptophan; (**B**) serotonin-to-tryptophan ratio; (**C**) serotonin; (**D**) 5-hydroxyindoleacetic acid-to-serotonin ratio; (**E**) 5-hydroxyindoleacetic acid.
